# Depletion of *Blautia wexlerae* and *Parabacteroides distasonis* in adiposity-related prehypertension

**DOI:** 10.3389/fmicb.2026.1873803

**Published:** 2026-06-29

**Authors:** Li Luo, Kejia Cheng, Bangwei Chen, Yaxin Li, Lei Ruan, Zhiming Li, Shida Zhu, Lijian Zhao, Cuntai Zhang, Yong Liu, Tao Li

**Affiliations:** 1 Center of Forensic Expertise, Affiliated Hospital of Zunyi Medical University, Zunyi, China; 2 BGI Genomics, Shenzhen, China; 3 Department of Pharmacology, Joint Laboratory of Guangdong-Hong Kong Universities for Vascular Homeostasis and Diseases, School of Medicine, Southern University of Science and Technology, Shenzhen, China; 4 Department of Geriatrics, Tongji Hospital, Tongji Medical College, Huazhong University of Science and Technology, Wuhan, China; 5 College of Life Sciences, University of Chinese Academy of Sciences, Beijing, China; 6 BGI Research, Shenzhen, China

**Keywords:** adiposity, *Blautia wexlerae*, microbiota, *Parabacteroides distasonis*, prehypertension

## Abstract

**Background:**

Prehypertension is more likely to develop into hypertension in individuals with adiposity. We aimed to explore how adiposity influences prehypertension through gut microbiota.

**Methods:**

Kaplan–Meier and Cox proportional hazard regression models were employed to evaluate the association between prehypertension and adiposity in 649 individuals. Among them, 197 consented to provide fecal samples and were divided, along with 184 additional participants, into healthy controls (HC), individuals with adiposity and normal tension (Ad-NT), and those with prehypertension (Ad-pHT) based on body mass index (BMI) and blood pressure. Shotgun metagenomic sequencing was performed on fecal samples, followed by taxonomic and functional annotations using MetaPhlAn and HUMAnN. Linear discriminant analysis effect size (LEfSe) was used to analyze differences in microbial species and metabolic pathways across groups. Partial Spearman rank correlation analysis was used to assess microbial interactions, and the relationships among metabolic pathways, species, BMI, and blood pressure.

**Results:**

Elevated BMI independently predicted the risk of prehypertension (adjusted HR = 1.072, 95% CI: 1.002–1.147). We observed the depletion of *Blautia wexlerae* and *Parabacteroides distasonis* in populations with Ad-pHT. A multiclass logistic regression model distinguished individuals with Ad-pHT from HC and those with adiposity and normal tension (Ad-NT) (AUC = 0.704). Microbiota-microbiota interactions gradually become complex from HC to Ad-NT to Ad-pHT groups. *Blautia wexlerae* and *Parabacteroides distasonis* were associated with pathways involved in carbohydrate degradation (PWY-8004), fermentation (ANAEROFRUCAT-PWY), biosynthesis of secondary metabolites (PWY-6270), amino acid (ARGININE-SYN4-PWY), quinol and quinone (PWY-7992), and nucleoside and nucleotide (PWY-6700).

**Conclusion:**

Shifts in *Blautia wexlerae* and *Parabacteroides distasonis*, as well as their relationships with pathways (energy metabolism and amino acid biosynthesis), were observed in adiposity-related prehypertension. *Blautia wexlerae* and *Parabacteroides distasonis* might represent promising candidates for next-generation probiotics targeting weight management and blood pressure reduction, which require validation in clinical studies.

## Background

Five modifiable risk factors (hypertension, obesity, non-high-density lipoprotein cholesterol, current smoking, and diabetes) could reduce the global burden of cardiovascular diseases (CVD) by approximately 50% (Global Cardiovascular Risk Consortium et al., 2023). In 2025, the Global Cardiovascular Risk Consortium proposed that modifying hypertension from present to absent during midlife was related to the most additional life-years free of CVD (Global Cardiovascular Risk Consortium et al., 2025). Prehypertension is considered a critical stage for intervention to prevent the development of hypertension and subsequent CVD (Mancia et al., 2023). Hence, people with prehypertension should receive intervention and treatment as soon as possible.

Young and middle-aged adults with general adiposity (overweight and obesity) are associated with an increased risk of prehypertension and hypertension (Tu et al., 2025; Yuan et al., 2022). Although the underlying mechanisms between adiposity and hypertension (such as insulin resistance and vascular dysfunction) have been demonstrated, their relative contributions, hierarchical interactions, and patient-specific predominance remain poorly defined (Clayton et al., 2023). Therefore, addressing the mechanisms behind adiposity with prehypertension can potentially alleviate the increase in the prevalence of hypertension and its adverse effects on health.

The gut microbiota is pivotal in maintaining general health (Parizadeh and Arrieta, 2023), and has become a hotspot in the pathogenesis of adiposity or hypertension. Patients with low blood pressure presented increased abundances of *Roseburia* spp. and *Ruminococcaceae* spp. (Verhaar et al., 2020). The abundance of *Butyricimonas* increased in patients with prehypertension and gradually decreased as hypertension developed. *Akkermansia muciniphila* (Depommier et al., 2019) and *Parabacteroides distasonis* (Wang et al., 2019) have been demonstrated to have inhibitory effects on obesity. Hence, the relationships between gut microbiota and obesity as well as hypertension might explain the connection between these two diseases. Guevara-Cruz et al. (2019) demonstrated that high body mass index (BMI) was closely associated with gut microbiota dysbiosis and elevated blood pressure. They also indicated that lifestyle intervention could not only improve metabolic syndrome and obesity, but also increase the abundance of *Akkermansia muciniphila* and *Faecalibacterium prausnitzii*, thereby reducing the risk of CVD. Zecheng et al. (2023) concluded that fecal microbiota transplantation could alleviate blood pressure and improve inflammatory responses in individuals with adiposity. Another study showed that gut dysbiosis was a shared mechanism in obesity and hypertension, which opened up new avenues for potential therapeutic interventions of gut microbiota to manage obesity-related hypertension (Belancic et al., 2026). Overall, these findings indicate that gut microbiota may play a role in the progression of coexisting adiposity and hypertension.

Here, we aimed to explore the relationships among the gut microbiota, adiposity, and prehypertension. Moreover, we attempted to explain the potential mechanisms by which gut microbiota regulated blood pressure in individuals with adiposity and prehypertension. Through comprehensive research and analysis, we found that *Blautia wexlerae* and *Parabacteroides distasonis* might promote weight loss by influencing pathways of energy metabolism and amino acid biosynthesis, which is associated with reduced blood pressure.

## Methods

### Definition of adiposity and prehypertension

BMI was classified into normal weight (18.5 ≤ BMI < 24) and adiposity (BMI ≥ 24). Blood pressure (SBP and DBP) was measured by a validated automated device (Omron, Kyoto, Japan). Prior to the measurement, the participants were instructed to rest for more than 5 min. Trained technicians placed a pressure cuff on the right arm. Two or more office visits were conducted for each participant. The average blood pressure was used for further analysis. According to the clinical practice guidelines for the management of hypertension in China, prehypertension is defined as SBP of 130–140 mmHg and/or DBP of 80–90 mmHg (Chinese Society of Cardiology, Chinese Medical Association et al., 2024).

### Study design and participant requirements

**Figure 1:**
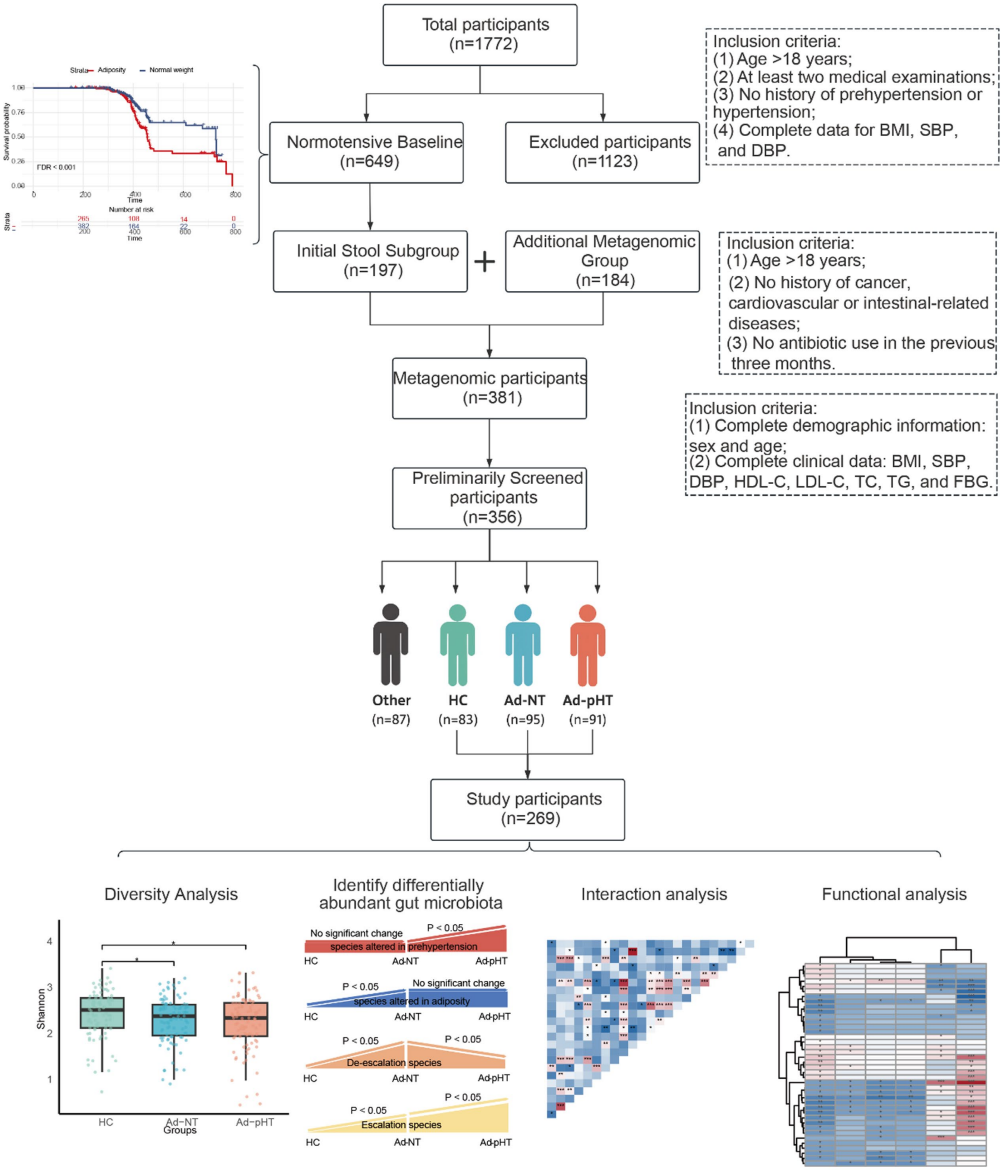
The study population and analytical workflow. BMI, body mass index; SBP, systolic blood pressure; DBP, diastolic blood pressure; TC, total cholesterol; HDL-C, high-density lipoprotein cholesterol; LDL-C, low-density lipoprotein cholesterol; TG, triglycerides; FBG, fasting blood glucose; HC, healthy controls; Ad-NT, adiposity and normal tension; Ad-pHT, adiposity and prehypertension.

This study employed a retrospective cohort design with a nested cross-sectional analysis. The conceptual framework of this study is shown in Figure 1. For the retrospective cohort design, a total of 4,656 adults (≥ 18 years) were recruited from annual physical examinations between April 2019 and December 2021 at Tongji Medical College, Huazhong University of Science and Technology. Moreover, 62 individuals with missing values for blood pressure (SBP and DBP), age, and sex were removed. Among these, 2,822 had only one examination were excluded. Therefore, a total of 1,772 participants who underwent at least two examinations were recruited. To ensure that all participants were free of prehypertension/hypertension at baseline and to assess incident prehypertension during follow-up, we excluded 1,122 participants who had been determined as having prehypertension/hypertension before the first recorded physical examination. Furthermore, one person with missing values of total cholesterol (TC), low-density lipoprotein cholesterol (LDL-C), high-density lipoprotein cholesterol (HDL-C), and triglycerides (TG) were removed. Consequently, 649 normotensive participants with at least two examinations were included to analyze the association between BMI and prehypertension (Supplementary Figure 1). The first examination confirming normotension (SBP or DBP below the thresholds for prehypertension and hypertension, respectively) was defined as baseline. Follow-up was extended to the first detection of incident prehypertension/hypertension or the last available examination before December 31, 2021, whichever occurred first. However, only 197 out of 649 people consented to provide fecal samples at the end of the retrospective cohort.

To ensure sufficient statistical power and minimize potential selection bias, we independently recruited an additional 184 participants from same hospital. In total, 381 participants (197 from the retrospective cohort and 184 additionally recruited) underwent fecal sampling for the nested cross-sectional metagenomic analysis. All these volunteers met the following inclusion criteria: (1) age > 18 years old, (2) no cancer, cardiovascular, or intestinal-related diseases, and (3) no record of antibiotic usage in the previous 3 months. After excluding those with missing demographic or clinical data, 356 participants were included. Based on their BMI and blood pressure measured at the time of fecal collection, participants were categorized into four groups: healthy controls (HC, *n* = 83), individuals with adiposity and normal tension (Ad-NT, *n* = 95), individuals with both adiposity and prehypertension (Ad-pHT, *n* = 91), and those with other phenotype combinations (others, *n* = 87). Considering the study focus on adiposity and prehypertension, the “others” group were excluded.

### Sample collection

Fecal samples from HC, Ad-NT, and Ad-pHT groups were stored in the MGIEasy Stool Sample Collection Kit (MGI, Shenzhen, China) after being gathered by participants at home and placed in the freezer (−20 °C). The fecal samples were subsequently placed on dry ice and transferred to the laboratory. Aliquots were then made and stored at −80 °C until DNA extraction. Blood samples were analyzed at a licensed clinical laboratory to measure fasting blood glucose (FBG), TC, TG, LDL-C, and HDL-C. Demographic information (sex, age, height, weight) was collected via a questionnaire. This study was approved by the Ethics Committees of Tongji Medical College, Huazhong University of Science and Technology (2020S146). Written informed consent was obtained from all participants before enrollment.

### Shotgun metagenomics sequencing of gut microbiota

Total genomic DNA was extracted from fecal samples using the MGIEasy Fecal Genomic DNA Extraction Kit (MGI, Shenzhen, China). The purity of DNA was determined using the A260/A280 ratio, and DNA quality was assessed using agarose gel electrophoresis. Whole-metagenome sequencing libraries were prepared from 500 ng of high-quality DNA using the MGIEasy DNA Library Preparation Kit (MGI, Shenzhen, China). Sequencing of 100-bp single-end reads was performed on the DNBSEQ-T10 × 4 platform. Low-quality reads and contaminating human reads were removed using SOAPnuke v2.1.7 (Chen et al., 2018) and Bowtie2 v2.5.0 with default parameters (Langmead and Salzberg, 2012) (reference database: GRCh38), respectively. The reads were analyzed using the bioBakery 3 tools (Beghini et al., 2021) that include MetaPhlAn v3.0.13 (reference database: mpa_v30_CHOCOPhlAn_201901) for taxonomic analysis and HUMAnN v3.1.1 for MetaCyc pathway annotation. To deal with sparse microbial data in all downstream analysis, we focused on species/pathways with a mean relative abundance ≥ 0.005% and a prevalence ≥ 10% in any of the three groups (HC, Ad-NT, and Ad-pHT) (Dong et al., 2023; Bokulich et al., 2013).

### Statistical analysis

All statistical analyses were conducted using R v4.5.1 (R Development Core Team, 2009). To evaluate the baseline characteristics among the three groups (HC, Ad-NT, and Ad-pHT), a comprehensive statistical analysis was performed. The overall *p* value was calculated using One-way ANOVA (BMI, DBP, LDL-C, TC) or Kruskal-Wallis test (age, SBP, TG, HDL-C, FBG) for continuous variables based on data normality. Additionally, pairwise independent *t*-tests or Wilcoxon rank-sum test were used for post-hoc comparisons between any two groups. Categorical variables were evaluated using the pairwise Chi-square test. To account for multiple comparisons and rigorously control the false positive rate, all *p*-values from pairwise post-hoc analyses were adjusted using the Benjamini-Hochberg False Discovery Rate (FDR) method (Benjamini and Hochberg, 2018).

Kaplan–Meier survival curves for prehypertension probability were compared across different BMI groups via the Wilcoxon rank-sum test with FDR adjustment. Cox proportional hazard regression models were employed to estimate hazard ratios (HR) and 95% confidence intervals (CI) using the Wald test with FDR adjustment (Prentice and Zhao, 2021). The multivariable analysis was adjusted for age, sex, lipid markers, diabetes, and hyperlipidemia.

Alpha diversity was calculated using the Shannon index and species richness by vegan package (Oksanen et al., 2024). Beta diversity was measured using Bray–Curtis dissimilarity and visualized through principal coordinates analysis (PCoA). Differences in microbial community structure were tested using permutational multivariate analysis of variance (PERMANOVA) with 10,000 permutations. To identify the enterotypes of the study cohort, we performed clustering analysis based on genus abundance. Prior to clustering, raw microbial counts were normalized using total sum scaling to account for variations in sequencing depth. To ensure all genus contribute equally to the clustering process, the normalized data were subjected to z-score standardization. Enterotypes were defined using the K-means clustering algorithm. The optimal number of clusters was determined using the Calinski-Harabasz index (Arumugam et al., 2011). To identify the driving genera of each enterotype, random forest analysis with ten-time fivefold cross-validation was performed using the randomForest() in R v4.5.1. Comparisons across groups were performed using the Wilcoxon rank-sum test with FDR adjustment.

To identify differentially abundant microbial taxa and pathways, linear discriminant analysis effect size (LEfSe) was applied (LDA > 2.0, *p* < 0.05) (Khleborodova et al., 2024; Gao et al., 2024). To evaluate the discriminatory power of microbial species, a one-vs-rest (OvR) logistic regression model using glm() in R v4.5.1 was constructed. Participants were divided into training set and testing set with a ratio of 8:2. Receiver operating characteristic (ROC) curves were generated and the areas under the curve (AUC) were calculated using pROC package (Nahm, 2022). Finally, the AUC of the model was calculated according to the sex subgroup in testing set.

Partial Spearman rank correlation analysis, adjusting for sex, age, BMI, SBP, DBP, HDL-C, TG, and FBG (which differed significantly among the three groups), was used to investigate microbial interactions and the relationships among differential metabolic pathways, *Blautia wexlerae*, *Parabacteroides distasonis*, BMI, and blood pressure. Additionally, co-occurrence interactions between species and pathways were assessed within each group. Student’s *t*-test for partial correlation was employed to compare distances among groups, and the Benjamini-Hochberg FDR was applied for multiple testing adjustment. FDR < 0.1 was set as the threshold for statistical significance to screen robust correlations between the variables of interest. Network construction and visualization were performed using the igraph v1.6.0 (Csárdi et al., 2024).

## Results

### BMI increases the risk of prehypertension

A total of 649 participants with an average age of 48 years, 471 males, were included. The median follow-up time for the entire cohort was 391 days. Among all participants, 266 were defined as having adiposity. SBP and DBP were significantly different (*p* < 0.001) between individuals with normal weight and those with adiposity. Moreover, prehypertension was more common in people with adiposity. The baseline characteristics of the participants are presented in Supplementary Table 1.

**Figure 2:**
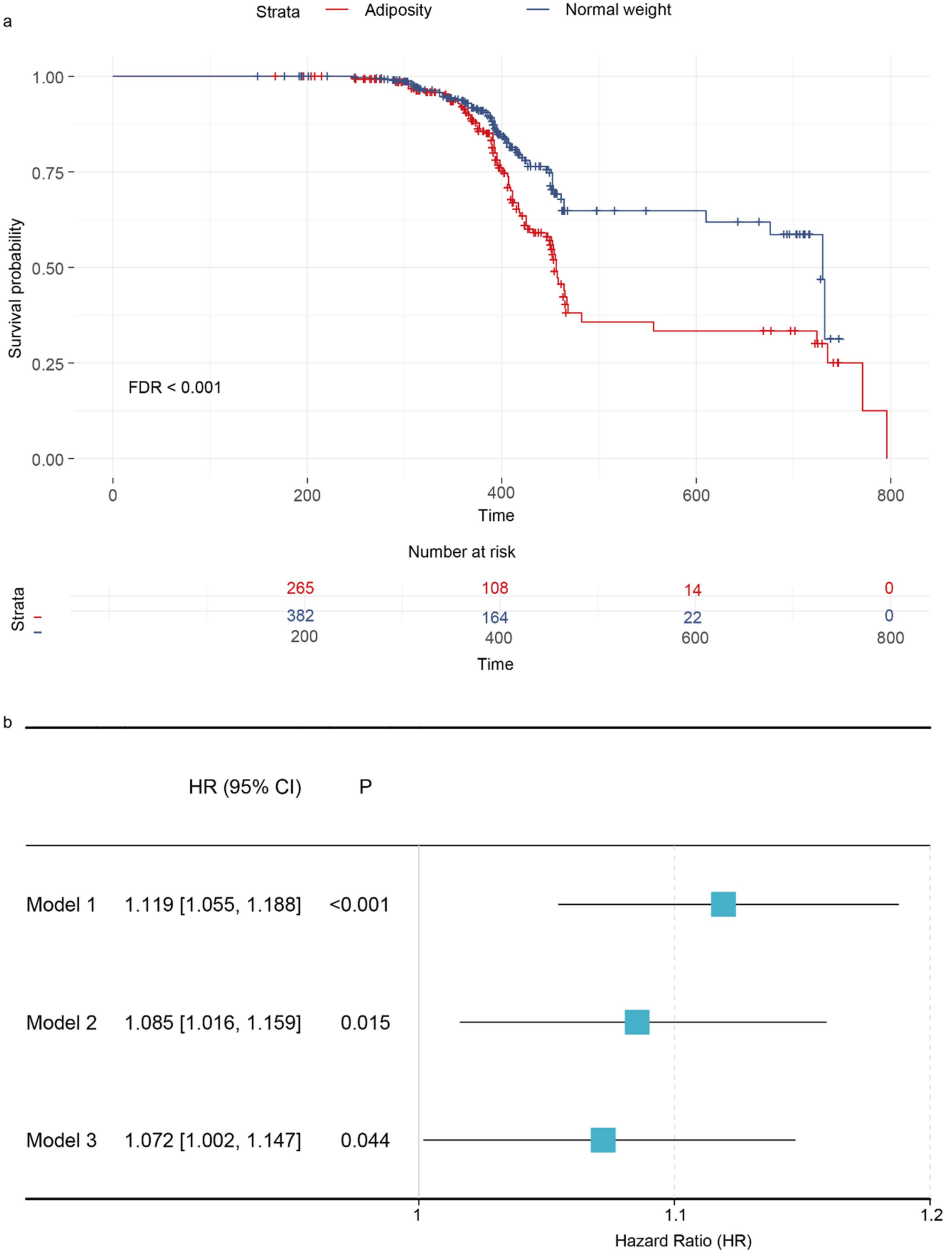
Association between body mass index (BMI) and the risk of prehypertension. **(a)** Kaplan–Meier survival analyses were compared via the Wilcoxon rank-sum test, with FDR adjustment (FDR < 0.001). **(b)** Cox regression analyses were compared using the Wald test, with FDR adjustment. Model 1: Univariate model. Model 2: Adjusted for age and sex. Model 3: Adjusted for age, sex, lipid profiles (total cholesterol, low-density lipoprotein cholesterol, high-density lipoprotein cholesterol), and diseases (diabetes and hyperlipidemia). HR, hazard ratio. CI, confidence intervals.

To investigate whether BMI was a potential risk factor for the development of prehypertension, a survival analysis was conducted. The Kaplan–Meier curve revealed that the probability of prehypertension in different BMI groups varied with time (FDR < 0.001, Figure 2a). Cox proportional hazards regression further confirmed that elevated BMI was associated with an increased risk of prehypertension (HR = 1.119, 95% CI: 1.055–1.188; Figure 2b). After adjusting for age, sex, lipid profiles, and diseases, the association remained significant (HR = 1.072, 95% CI: 1.002–1.147).

### Overview of the gut microbiota

To gain insight into the composition and alteration of the gut microbiota in populations with adiposity and prehypertension, fecal samples from 83 HC, 95 participants with Ad-NT, and 91 individuals with Ad-pHT were sequenced. The characteristics of the participants are listed in Table 1. Compared with the HC group, the Ad-pHT and Ad-NT groups had more males, higher age, and greater BMI. Except for TC and LDL-C, all differences between groups were statistically significant (FDR < 0.001). Moreover, no statistically significant differences in blood pressure (SBP and DBP) were observed between the HC and Ad-NT groups, nor were there any differences in TG and FBG between the Ad-pHT and Ad-NT groups.

**Table 1:** The characteristics of the study participants.

Characteristics	Total (*n* = 356)	HC (*n* = 83)	Ad-NT (*n* = 95)	Ad-pHT (*n* = 91)	Overall *p* value
Sex, Male, *n* (%)	301(84.55)	60(72.29)	86(90.53)	83(91.21)	
Age (years)	46 ± 9.95	41.39 ± 9.77	44.67 ± 9.52	47.96 ± 8.87^b***^	<0.001
BMI (kg/m^2^)	24.93 ± 2.86	21.85 ± 1.33^a***^	25.77 ± 1.58 ^c***^	26.74 ± 2.12 ^b***^	<0.001
SBP (mmHg)	125.24 ± 15.9	113.46 ± 9.01	113.96 ± 8.13^c***^	132.41 ± 9.29^b***^	<0.001
DBP (mmHg)	78.73 ± 10.99	70.59 ± 6.01	70.48 ± 5.35 ^c***^	84.2 ± 5.92 ^b***^	<0.001
TC (mmol/L)	4.61 ± 0.91	4.41 ± 0.86	4.66 ± 0.88	4.57 ± 0.87	0.166
HDL-C (mmol/L)	1.23 ± 0.29	1.34 ± 0.31 ^a***^	1.18 ± 0.24	1.13 ± 0.24 ^b***^	<0.001
LDL-C (mmol/L)	2.8 ± 0.81	2.57 ± 0.74	2.86 ± 0.79	2.75 ± 0.79	0.051
TG (mmol/L)	1.64 ± 1.09	1.32 ± 0.84	1.69 ± 1.07	1.98 ± 1.29 ^b***^	<0.001
FBG (mmol/L)	5.2 ± 1.24	4.74 ± 0.63	5.14 ± 1.51	5.51 ± 1.25 ^b***^	<0.001

Continuous variables were described as mean ± standard deviation and categorical variables were presented as *n* (percentages). BMI, body mass index; SBP, systolic blood pressure; DBP, diastolic blood pressure; TC, total cholesterol; HDL-C, high-density lipoprotein cholesterol; LDL-C, low-density lipoprotein cholesterol; TG, triglycerides; FBG, fasting blood glucose; The overall *p* value was calculated using One-way ANOVA or Kruskal-Wallis test for continuous variables based on data normality, and the Chi-square test for categorical variables. Pairwise group differences were evaluated with FDR adjustment. ^a^HC vs. Ad-NT; ^b^HC vs. Ad-pHT; ^c^Ad-NT vs. Ad-pHT. ***FDR < 0.001.

**Figure 3:**
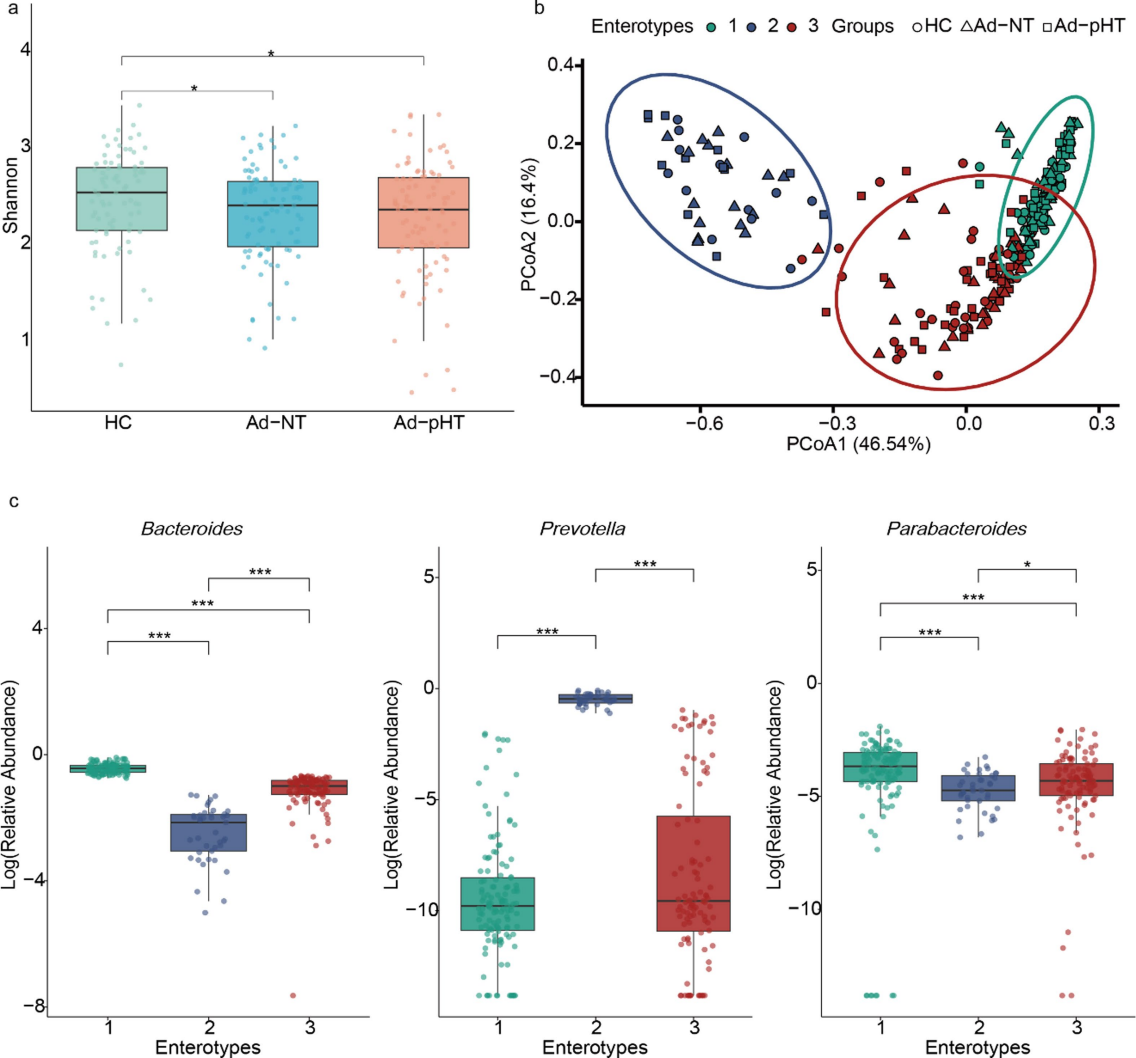
Gut microbiota diversity and enterotype classification. **(a)** Shannon diversity was compared using the Wilcoxon rank-sum test, with FDR adjustment (*), FDR < 0.05. **(b)** Principal coordinates analysis (PCoA) revealed three enterotypes, and group differences were assessed by PERMANOVA with FDR = 0.001. **(c)** Relative abundances of dominant genera defining each enterotype were compared using the Wilcoxon rank-sum test with FDR adjustment (*), FDR < 0.05; (***), FDR < 0.001. HC, healthy controls; Ad-NT, adiposity and normal tension; Ad-pHT, adiposity and prehypertension.

Among all the populations, 15 phyla, 218 genera, and 649 species were obtained. The predominant phyla were *Bacteroidetes* and *Firmicutes* (Supplementary Figure 2a). The genus *Bacteroides* was dominant in the Ad-pHT group, whereas *Prevotella* was most abundant in the Ad-NT group (Supplementary Figure 2b). Among the top 10 genera, only significant variations were observed for *Escherichia* and *Eubacterium eligens* when HC was compared with the Ad-NT group (FDR < 0.1, Supplementary Figures 2b,c). No significant differences in richness or beta diversity were observed, except for richness between HC and Ad-pHT groups (FDR < 0.05, Supplementary Figures 2d,e). In addition, the Shannon diversity index differed significantly between HC and Ad-pHT groups, as well as between HC and Ad-NT group (FDR < 0.05, Figure 3a). To explore the differences in microbial communities across disease stages, enterotypes were identified based on genus abundance. Enterotype 1, enterotype 2, and enterotype 3 were characterized by *Bacteroides*, *Prevotella*, and *Parabacteroides*, respectively (Figures 3b,c). Enterotype 1 predominated in all three groups (43.4% in HC, 48.4% in participants with Ad-NT, and 50.5% in participants with Ad-pHT), followed by enterotype 3 (41.0, 34.7, and 36.3%, respectively) and enterotype 2 (15.7, 16.8, and 13.2%, respectively). The results indicated that the composition and structure of gut microbiota were similar among the three groups.

### Gut microbiota alterations in prehypertension with adiposity

**Figure 4:**
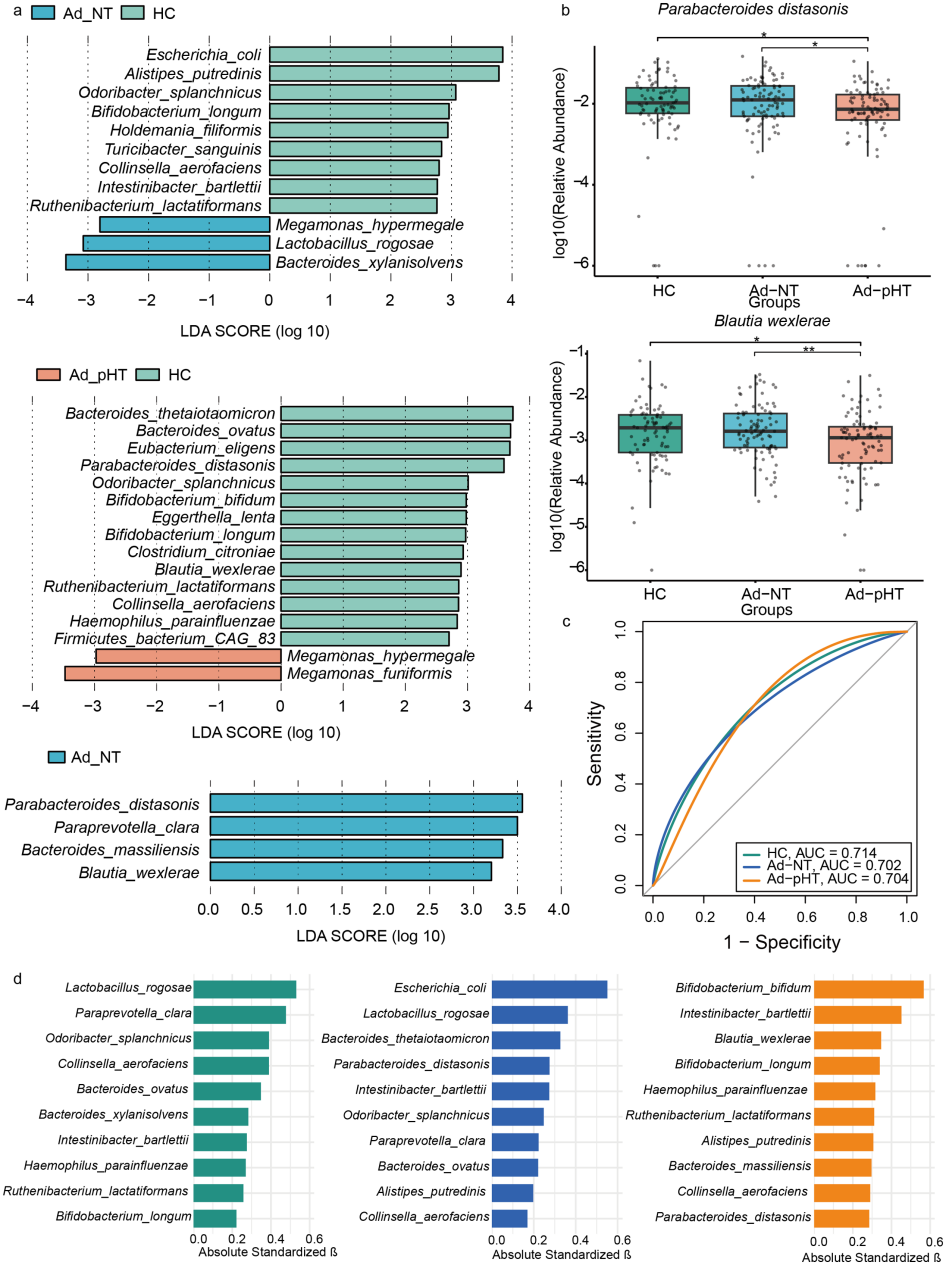
Differential gut microbial species and their predictive performance. **(a)** Different species across three groups determined by LEfSe (LDA > 2, *p* < 0.05). **(b)** The relative abundances of two species among 3 group were compared using Wilcoxon rank-sum test with FDR adjustment (*), FDR < 0.05; (**), FDR < 0.01. **(c)** AUC of the multiclass model with 25 species. **(d)** Importance of top 10 features in the models. The green bar shows the absolute standardized *β* in the model for HC, while the blue bar is for Ad-NT and the orange for Ad-pHT. Species highlighted in red were negatively associated with prehypertension, whereas those in blue were altered in adiposity. HC, healthy controls; Ad-NT, adiposity and normal tension; Ad-pHT, adiposity and prehypertension.

A total of 25 species were significantly different across three groups by LEfSe analysis (LDA > 2, *p* < 0.05, Figure 4a). Compared with those in the HC group, the abundances of *Bifidobacterium longum*, *Odoribacter splanchnicus*, *Collinsella aerofaciens*, and *Ruthenibacterium lactatiformans* in the participants with Ad-NT and Ad-pHT were lower, and *Megamonas hypermegale* was enriched. However, there were no significant changes in these five species between individuals with Ad-NT and Ad-pHT, suggesting that their observed differences from HC might be driven by adiposity rather than prehypertension. Consistency of the results was confirmed through further testing using the Wilcoxon rank-sum test with Benjamini-Hochberg FDR adjustment (Supplementary Figure 3). To identify species specifically associated with the transition from adiposity to prehypertension, we selected species that showed significant differences in both comparisons (Ad-pHT vs. HC and Ad-pHT vs. Ad-NT), and consistent directional changes. Among the 25 differential species, only *Blautia wexlerae* and *Parabacteroides distasonis* met these stringent criteria (Figure 4b). We inferred that these two species might be specifically associated with prehypertension in the context of adiposity.

Considering the effect of sex on gut microbiota, sex-associated differentially abundant species were identified by LEfSe. Sex mainly affected the species associated with adiposity (*Megamonas hypermegale*, *Bifidobacterium longum*, and *Ruthenibacterium lactatiformans*), but not those associated with prehypertension (Supplementary Figure 4a). Furthermore, we performed a multiclass logistic regression model using 25 differential species to distinguish three groups. The AUC values were 0.714 for HC, 0.702 for people with Ad-NT, and 0.704 for participants with Ad-pHT, respectively (Figure 4c). Due to a high imbalance between sexes, we further analyzed the sex subgroups in the testing set. The AUC values for males and females, respectively, were 0.720 and 0.680 for HC, 0.667 and 0.714 for Ad-NT, and 0.750 and 0.625 for Ad-pHT (Supplementary Figure 4b). These findings suggested that the model performed similarly across sexes. Among the top 10 features of the model, *Blautia wexlerae* was a key discriminatory factor for the Ad-pHT group. Additionally, *Parabacteroides distasonis* was significantly associated with both Ad-NT and Ad-pHT groups (Figure 4d). These findings suggested that the depletion of *Blautia wexlerae* and *Parabacteroides distasonis* might be associated with adiposity-related prehypertension.

### Microbiota-microbiota interaction shifts in prehypertension with adiposity

**Figure 5:**
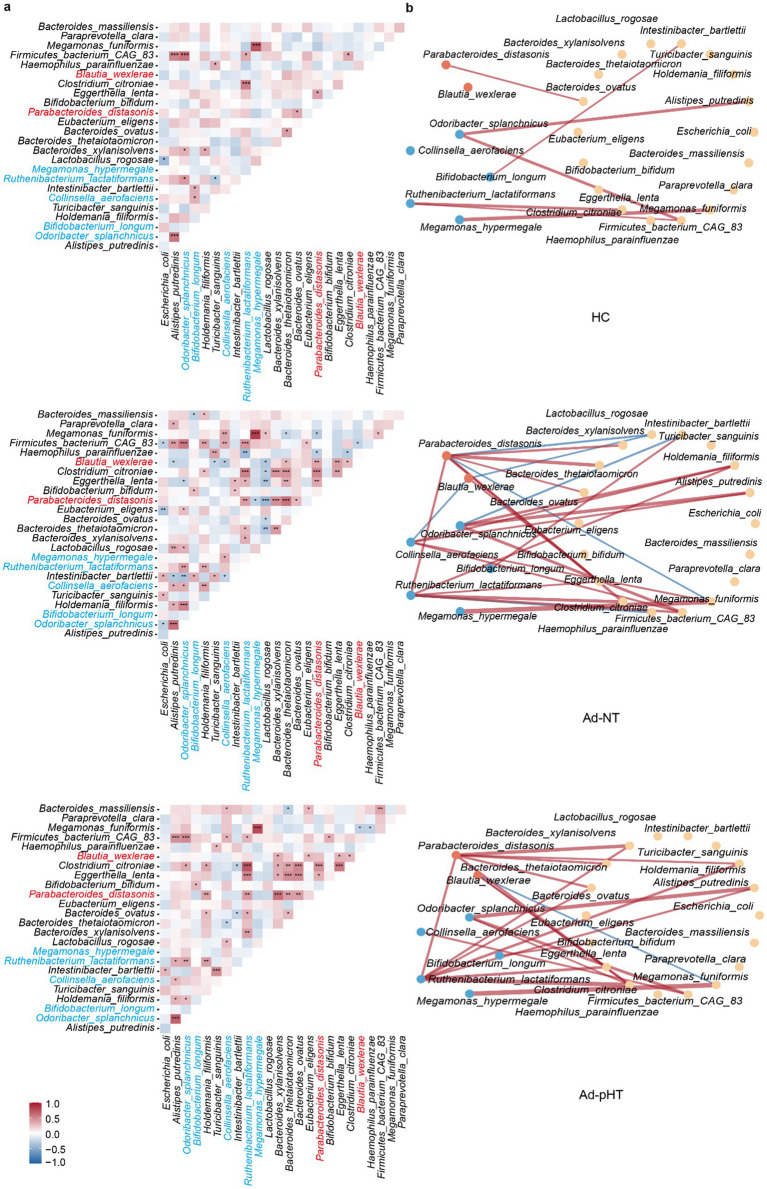
Microbiota-microbiota interactions in HC, Ad-NT, and Ad-pHT. **(a)** Partial Spearman rank correlation analysis among 25 species were shown by heatmap. The partial correlation coefficients and corresponding significance were calculated using partial correlation analysis, strictly adjusting for sex, age, BMI, SBP, DBP, HDL-C, TG and FBG. *p*-values were corrected for multiple comparisons using the False Discovery Rate (FDR) method. Asterisks indicate statistical significance after FDR adjustment: *, FDR < 0.1; **, FDR < 0.01; ***, FDR < 0.001. Species highlighted in red were negatively associated with prehypertension, while those in blue were altered in adiposity. **(b)** Correlation network showing microbial correlations in HCs, Ad-NT, and Ad-pHT. Correlations were calculated using partial correlation coefficients with criteria of absolute rho > 0.3 and FDR adjustment FDR < 0.05. The red nodes were species negatively associated with prehypertension, the blue nodes were species altered in adiposity, and the yellow nodes were other species.

To assess the interactions among differential species, partial Spearman rank correlation analysis was conducted across HC, participants with Ad-NT, and participants with Ad-pHT. Microbial networks gradually became complex from HC to Ad-NT to Ad-pHT (Figure 5a). Correlations between *Blautia wexlerae* and *Eggerthella lenta* emerged in participants with Ad-NT (rho = 0.400, FDR = 0.002) and Ad-pHT (rho = 0.304, FDR = 0.047), but were absent in HC. Additionally, *Blautia wexlerae* exhibited positive correlations with *Clostridium citroniae* (rho = 0.285, FDR = 0.068) and negative correlation with *Megamonas funiformis* (rho = −0.281, FDR = 0.072) in the Ad-pHT group. *Parabacteroides distasonis* was positively correlated with *Bacteroides ovatus* across all groups (HC: rho = 0.360, FDR = 0.044; Ad-NT: rho = 0.291, FDR = 0.035; Ad-pHT: rho = 0.404, FDR = 0.003). Notably, a negative relationship was observed between *Megamonas funiformis* (a well-known adiposity-associated species) and *Parabacteroides distasonis* in participants with Ad-NT (rho = −0.323, FDR = 0.016).

Additionally, interaction networks were constructed using species that were differentially abundant in individuals with adiposity or altered in participants with prehypertension (absolute rho > 0.3 and FDR < 0.05, Figure 5b). No significant co-occurrence relationships were observed in the HC group. *Parabacteroides distasonis* was associated with *Ruthenibacterium lactatiformans* in individuals with Ad-NT and Ad-pHT. Moreover, participants with Ad-NT had more links. Interestingly, the links between *Collinsella aerofaciens* and *Blautia wexlerae* in individuals with Ad-NT were diminished in participants with Ad-pHT. These results suggested that ecological interactions among gut species might be altered in prehypertension with adiposity.

### Microbial functional alterations in prehypertension with adiposity

A total of 330 pathways with a prevalence ≥ 10% and an average relative abundance ≥ 0.005% were included. No differences were observed in richness or beta diversity; however, Shannon diversity differed significantly (Supplementary Figure 5a). Moreover, we identified 38 differentially abundant metabolic pathways between HC and participants with Ad-pHT (Supplementary Figure 5b). These pathways were primarily involved in amino acid metabolism, carbohydrate degradation, and secondary metabolites biosynthesis. Amino acid metabolism pathways, including ARGININE-SYN4-PWY (L-ornithine biosynthesis II), PWY-2942 (L-lysine biosynthesis III), and HSERMETANA-PWY (L-methionine biosynthesis III), were upregulated in the Ad-pHT group, suggesting potential alterations in nitrogen balance and biosynthetic processes. Interestingly, vitamin biosynthesis pathways (PANTO-PWY, 1CMET2-PWY, and PANTOSYN-PWY) were enriched only in participants with the Ad-pHT, whereas fermentation-related pathways (PWY-7383 and ANAEROFRUCAT-PWY) were downregulated in this group. Additionally, PWY-8004 (Entner-Doudoroff pathway I) and PWY-6270 (Isoprene biosynthesis I) were decreased in individuals with Ad-pHT. To investigate the functional differences associated with species alterations in prehypertension or adiposity, microbial pathway enrichment analysis was carried out (Supplementary Figure 6). These species contributed to 24 differential pathways, among which alterations in participants with prehypertension were more significant than those in participants with adiposity alone. These findings suggested distinct metabolic shifts associated with prehypertension in the context of adiposity, which might reflect underlying disruptions in host-microbiome interactions and metabolic homeostasis.

### Metabolic disruptions driven by key gut microbiota

**Figure 6:**
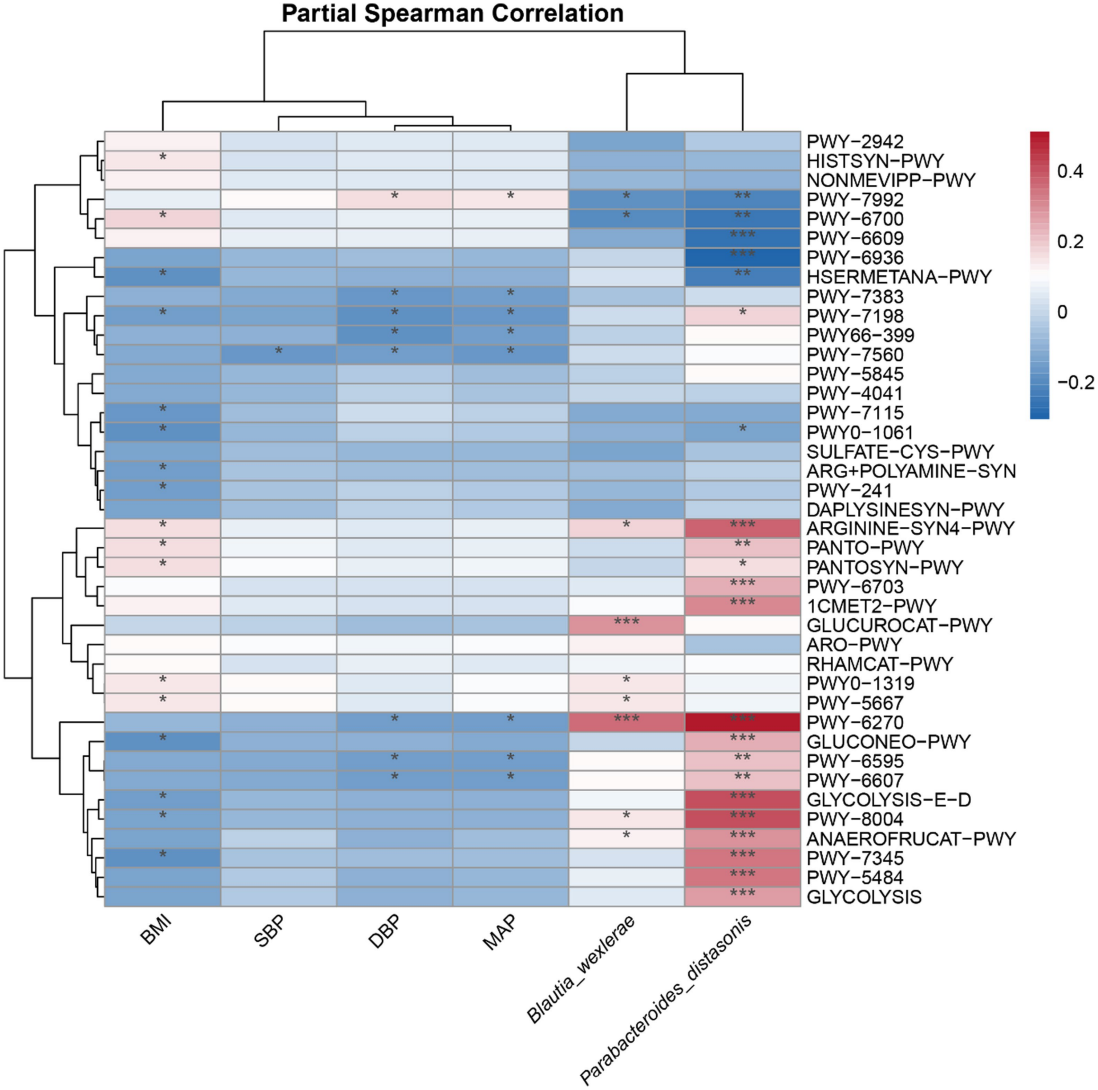
The interaction between key species and differential pathways, as well as their relationships with BMI and blood pressure. The hierarchical clustering heatmap illustrates pairwise correlations between 40 differential metabolic pathways (rows) and 6 target variables (columns; including 2 key bacterial species, BMI, SBP, DBP, and MAP). Partial correlation coefficients and corresponding significance were calculated using partial correlation analysis, strictly controlling for sex, age, TG, HDL-C, and FBG. *p*-values were adjusted across the matrix using the False Discovery Rate (FDR) method to control for multiple comparisons. Asterisks printed within the cells denote statistical significance after strict FDR correction: *FDR < 0.1; **FDR < 0.01; ***FDR < 0.001. BMI, body mass index; SBP, systolic blood pressure; DBP, diastolic blood pressure; MAP, mean arterial pressure.

To elucidate the potential mechanisms underlying the interaction between adiposity and prehypertension, partial Spearman rank correlation (adjusting for sex, age, HDL-C, LDL-C, TG, and FBG) between species altered in adiposity-related prehypertension (*Blautia wexlerae* and *Parabacteroides distasonis*), differential pathways, and clinical indices (BMI and blood pressure) were evaluated (Figure 6). *Blautia wexlerae* was significantly correlated with 8 pathways, while *Parabacteroides distasonis* was associated with 20 pathways. Six pathways were shared between both species, suggesting potential functional redundancy or cooperative interactions. These shared pathways included carbohydrate degradation (PWY-8004), fermentation (ANAEROFRUCAT-PWY), biosynthesis of secondary metabolites (PWY-6270), amino acid (ARGININE-SYN4-PWY), quinol and quinone (PWY-7992), and nucleoside and nucleotide (PWY-6700). Reduced abundance of *Blautia wexlerae* and *Parabacteroides distasonis* was associated with depletion of ARGININE-SYN4-PWY and enrichment of PWY-6700. Moreover, both pathways were positively associated with BMI (PWY-6700: rho = 0.170, FDR = 0.041; ARGININE-SYN4-PWY: rho = 0.152, FDR = 0.067). PWY-7992 was positively associated with blood pressure (DBP and MAP) but negatively correlated with *Blautia wexlerae* and *Parabacteroides distasonis*. In contrast, PWY-6270 showed the opposite pattern. PWY-8004 was positively correlated with *Blautia wexlerae* and *Parabacteroides distasonis* but negatively associated with BMI and blood pressure. We further examined the co-occurrence patterns between three common pathways (ARGININE-SYN4-PWY, PWY-6700, and PWY-8004) and species altered in adiposity and prehypertension (Supplementary Figure 7). *Parabacteroides distasonis* and *Megamonas hypermegale* were related to ARGININE-SYN4-PWY and PWY-8004 across all groups. Additionally, connections between adiposity-related species and pathways were absent in participants with Ad-pHT. Interestingly, the link between ARGININE-SYN4-PWY and *Collinsella aerofaciens* was diminished in the Ad-NT group. The associations implicated that the altered abundance of *Blautia wexlerae* and *Parabacteroides distasonis* in adiposity-related prehypertension might disrupt key metabolic pathways.

## Discussion

Our study provided insight into microbial composition and potential mechanisms underlying the association between adiposity and prehypertension. While BMI was the best anthropometric indicator for identifying prehypertension (Han et al., 2025), our findings suggested that this association might be partially mediated by specific microbial depletion rather than broad community restructuring. Notably, *Blautia wexlerae* and *Parabacteroides distasonis* were decreased in participants with Ad-pHT, resulting in reconstructed ecological interactions.

*Parabacteroides*, a genus known for carbohydrate metabolism and short-chain fatty acids (SCFA) production, was associated with adiposity and metabolic syndrome (Cui et al., 2022). *Parabacteroides distasonis*, a key species in human gut microbiota (Zhang W. et al., 2022), was previously reported to be negatively associated with obesity (Wang et al., 2019). However, the lack of difference between HC and those with Ad-NT in our study might be explained by differences in adiposity severity across study populations. *Parabacteroides distasonis* ferments dietary oligosaccharides to produce acetate and propionate (Lei et al., 2021). Jama et al. (2023) suggested that prebiotic acetylated and butyrylated high-amylose maize starch modulated SBP by increasing SCFA production and prevalence of *Parabacteroides distasonis*. *Ruthenibacterium lactatiformans*, a novel butyrate-related species (Becker et al., 2022), exhibited a robust correlation with fat metabolic indicators and alleviated obesity (Wu et al., 2024). The coexistence of *Ruthenibacterium lactatiformans* and *Parabacteroides distasonis* might suggest functional interactions associated with adiposity and prehypertension through SCFA-related pathways. Adiposity with prehypertension was associated with elevated vitamin biosynthesis pathways (PANTO-PWY, 1CMET2-PWY, and PANTOSYN-PWY), which were significantly positively correlated with BMI and *Parabacteroides distasonis*. Given that PANTO-PWY might help *Bacteroides vulgatus* resist obesity (Zhang F. et al., 2022), we hypothesized that *Parabacteroides distasonis* might have protective effects against adiposity and prehypertension through regulation of vitamin biosynthesis pathways.

*Blautia wexlerae* (genus *Blautia*) was less common in persons with Ad-pHT. Accumulating evidence suggests that *Blautia* is significantly reduced in the prehypertensive stage (Yu et al., 2025) or individuals with hypertension (Chang et al., 2020; Sun et al., 2025). A study involving 377 subjects demonstrated that *Blautia* was associated with parameters of the renin-angiotensin-aldosterone system, which is the core mechanism of endocrine regulation in hypertension (Mizoguchi et al., 2023). However, whether *Blautia wexlerae* impacts blood pressure remains unclear. Lv et al. reported that *Blautia wexlerae* was significantly enriched in healthy controls compared to hypertensive patients, suggesting a protective association with normal blood pressure (Ding et al., 2023). Hosomi et al. (2022) found that *Blautia wexlerae* improved obesity and diabetes via regulating amino acid metabolism and carbohydrate metabolism. Another study showed a significant reduction in *Blautia wexlerae* in obesity with insulin resistance, which was accompanied by higher proinflammatory cytokines (Benitez-Paez et al., 2020). Additionally, the co-occurrence of *Blautia wexlerae* and *Collinsella aerofaciens* (an obesity biomarker (Companys et al., 2021; Gallardo-Becerra et al., 2020)) was observed in participants with Ad-NT but diminished in participants with Ad-pHT. These ecological changes might indicate gut microbiota reorganization in prehypertension development, with *Blautia wexlerae* potentially involved in adiposity-related prehypertension via amino acid metabolism pathways.

*Blautia wexlerae* and *Parabacteroides distasonis* shared six pathways. ARGININE-SYN4-PWY, essential for the hydrolysis of L-arginine into L-ornithine (Wu et al., 2019), showed a positive relationship with both species. Its negative association with *Collinsella aerofaciens* was absent in participants with Ad-NT, suggesting that adiposity without prehypertension may disrupt this ecological association. Given that L-ornithine synthesis might regulate blood pressure by vascular NO production (Heuser et al., 2025), this disruption could have functional relevance. PWY-6700, involved in queuosine biosynthesis (a tRNA modification associated with cancer and diabetes) (Suzuki et al., 2025), correlated positively with BMI, consistent with the weight-gain effect *of Phyllanthus emblica* in chronic colonic inflammation (Li et al., 2023). Furthermore, relationships between PWY-6700 and adiposity-related species disappeared in participants with Ad-pHT, suggesting that changes in species-pathway associations accompany the transition from adiposity to prehypertension. The superpathway of menaquinol-8 biosynthesis I (vitamin K_2_) has been shown to be abundant in individuals with diabetes (Dash and Al Bataineh, 2021), implying a role in insulin resistance and inflammation. Since *Blautia wexlerae* and *Parabacteroides distasonis* were negatively correlated with PWY-7992 (another vitamin K_2_ biosynthesis pathway), their depletion could lead to vitamin K_2_ biosynthesis enrichment and consequent blood pressure increase. The populations with Ad-pHT had lower levels of ANAEROFRUCAT-PWY (homolactic fermentation) and PWY-8004 (Entner-Doudoroff Pathway), which were alternative pathways for catabolizing glucose to lactate and pyruvate (Law et al., 2024). Because lactate is the precursor metabolite for SCFA biosynthesis, low ANAEROFRUCAT-PWY activity may impair SCFA synthesis. Li et al. (2021) demonstrated that changes in ANAEROFRUCAT-PWY might contribute to obesity, consistent with our observation of decreased carbohydrate fermentation capacity in adiposity-related prehypertension. Pyruvate, a glycolytic product and precursor for fatty acid and amino acid synthesis, has been demonstrated to suppress obesity and adipose tissue inflammation through its interaction with cytoplasmic phospholipase A_2_ (Hasan et al., 2024). Collectively, *Blautia wexlerae* and *Parabacteroides distasonis* (potential next-generation probiotics) may promote vasodilation, inhibit adipogenesis, and reduce blood pressure by modifying these pathways (Tiwari et al., 2024; Rui et al., 2024; Vallianou et al., 2023), a finding that needs further validation in animal models.

Our study has several limitations. First, the modest sample size and male predominance might constrain specific correlation analyses, although findings were consistent with previous research. Second, dependence on a single time point limited the ability to track longitudinal changes in microbiota disruption during blood pressure progression. Third, the assessment of adiposity relied solely on BMI, future prospective studies should incorporate other adiposity measures (such as waist circumference, hip circumference, waist/hip ratio, and body fat percentage). Fourth, short read-based metagenomics approaches might be unable to identify non-bacterial components of the microbiome due to their dependence on reference databases. Fifth, although there was no significant difference in sex-adjusted model validation and partial Spearman correlation analysis, future studies should still consider differences between males and females. Finally, the effects of unmeasured confounders (such as diet, lifestyle, and medication) could not be ignored. Future large-scale, well-designed longitudinal studies are needed to validate and extend our findings.

## Conclusion

In conclusion, this study characterized the composition and functional potential of the gut microbiota in individuals with adiposity-related prehypertension. Shifts in *Blautia wexlerae* and *Parabacteroides distasonis*, as well as their relationships with pathways (energy metabolism and amino acid biosynthesis), were observed in the progression of prehypertension. *Blautia wexlerae* and *Parabacteroides distasonis* might represent promising candidates for next-generation probiotics targeting weight management and blood pressure reduction, which require validation in clinical studies.

## Data Availability

The raw sequence data reported in this paper have been deposited in the Genome Sequence Archive in National Genomics Data Center, China National Center for Bioinformation/Beijing Institute of Genomics, Chinese Academy of Sciences (GSA-Human: HRA009046), which are publicly accessible at: https://ngdc.cncb.ac.cn/gsa-human/browse/HRA009046.

## References

[ref1] Arumugam M. Raes J. Pelletier E. le Paslier D. Yamada T. Mende D. R. . (2011). Enterotypes of the human gut microbiome. Nature 473, 174–180. doi: 10.1038/nature09944, 21508958 PMC3728647

[ref2] Becker A. Schmartz G. P. Gröger L. Grammes N. Galata V. Philippeit H. . (2022). Effects of resistant starch on symptoms, fecal markers, and gut microbiota in Parkinson's disease - the RESISTA-PD trial. Genomics Proteomics Bioinformatics 20, 274–287. doi: 10.1016/j.gpb.2021.08.009, 34839011 PMC9684155

[ref3] Beghini F. McIver L. J. Blanco-Míguez A. Dubois L. Asnicar F. Maharjan S. . (2021). Integrating taxonomic, functional, and strain-level profiling of diverse microbial communities with bioBakery 3. eLife 10:e65088. doi: 10.7554/eLife.65088, 33944776 PMC8096432

[ref4] Belancic A. Fajkic A. Belančić A. Fajkić A. Sener Y. Z. Jelaković A. . (2026). Gut dysbiosis as a shared mechanism in obesity and hypertension: exploring a promising therapeutic avenue. Endocrinol. Diab. Metabol. 9:e70159. doi: 10.1002/edm2.70159 PMC13580870 41902532

[ref5] Benitez-Paez A. Gomez Del Pugar E. M. López-Almela I. Moya-Pérez Á. Codoñer-Franch P. Sanz Y. . (2020). Depletion of *Blautia* species in the microbiota of obese children relates to intestinal inflammation and metabolic phenotype worsening. mSystems 5:e00857. doi: 10.1128/mSystems.00857-19, 32209719 PMC7093825

[ref6] Benjamini Y. Hochberg Y. (2018). Controlling the false discovery rate: a practical and powerful approach to multiple testing. J. R. Stat. Soc. B Stat. Methodol. 57, 289–300. doi: 10.1111/j.2517-6161.1995.tb02031.x

[ref7] Bokulich N. A. Subramanian S. Faith J. J. Gevers D. Gordon J. I. Knight R. . (2013). Quality-filtering vastly improves diversity estimates from Illumina amplicon sequencing. Nat. Methods 10, 57–59. doi: 10.1038/nmeth.2276, 23202435 PMC3531572

[ref8] Chang Y. Chen Y. Zhou Q. Wang C. Chen L. Di W. . (2020). Short-chain fatty acids accompanying changes in the gut microbiome contribute to the development of hypertension in patients with preeclampsia. Clin. Sci. 134, 289–302. doi: 10.1042/CS20191253, 31961431

[ref9] Chen Y. Chen Y. Shi C. Huang Z. Zhang Y. Li S. . (2018). SOAPnuke: a MapReduce acceleration-supported software for integrated quality control and preprocessing of high-throughput sequencing data. Gigascience 7, 1–6. doi: 10.1093/gigascience/gix120, 29220494 PMC5788068

[ref10] Chinese Society of Cardiology, Chinese Medical Association; Hypertension Committee of Cross-Straits Medicine Exchange Association; Cardiovascular Disease Prevention and Rehabilitation Committee, Chinese Association of Rehabilitation Medicine (2024). Clinical practice guideline for the management of hypertension in China. Chin. Med. J. 137, 2907–2952. doi: 10.1097/CM9.0000000000003431 39653517 PMC11706600

[ref11] Clayton T. L. Fitch A. Bays H. E. (2023). Obesity and hypertension: obesity medicine association (OMA) clinical practice statement (CPS) 2023. Obes Pillars. 8:100083. doi: 10.1016/j.obpill.2023.100083, 38125655 PMC10728712

[ref12] Companys J. Gosalbes M. J. Pla-Pagà L. Calderón-Pérez L. Llauradó E. Pedret A. . (2021). Gut microbiota profile and its association with clinical variables and dietary intake in overweight/obese and lean subjects: a cross-sectional study. Nutrients 13:2032. doi: 10.3390/nu13062032, 34199239 PMC8231825

[ref13] Csárdi G. Nepusz T. Traag V. Horvát S. Zanini F. Noom D. . (2024) Igraph: network analysis and visualization in R. Available online at: https://CRAN.R-project.org/package=igraph

[ref14] Cui Y. Zhang L. Wang X. Yi Y. Shan Y. Liu B. . (2022). Roles of intestinal Parabacteroides in human health and diseases. FEMS Microbiol. Lett. 369:fnac072. doi: 10.1093/femsle/fnac072, 35945336

[ref15] Dash N. R. Al Bataineh M. T. (2021). Metagenomic analysis of the gut microbiome reveals enrichment of Menaquinones (vitamin K2) pathway in diabetes mellitus. Diabetes Metab. J. 45, 77–85. doi: 10.4093/dmj.2019.0202, 32431114 PMC7850878

[ref16] Depommier C. Everard A. Druart C. Plovier H. van Hul M. Vieira-Silva S. . (2019). Supplementation with *Akkermansia muciniphila* in overweight and obese human volunteers: a proof-of-concept exploratory study. Nat. Med. 25, 1096–1103. doi: 10.1038/s41591-019-0495-2, 31263284 PMC6699990

[ref17] Ding H. Xu Y. Cheng Y. Zhou H. Dong S. Wu J. . (2023). Gut microbiome profile of Chinese hypertension patients with and without type 2 diabetes mellitus. BMC Microbiol. 23:254. doi: 10.1186/s12866-023-02967-x, 37689641 PMC10492291

[ref18] Dong C. Guan Q. Xu W. Zhang X. Jin B. Yu S. . (2023). Disentangling the age-related manner in the associations between gut microbiome and women's health: a multi-cohort microbiome study. Gut Microbes 15:2290320. doi: 10.1080/19490976.2023.2290320, 38059752 PMC10730178

[ref19] Gallardo-Becerra L. Cornejo-Granados F. García-López R. Valdez-Lara A. Bikel S. Canizales-Quinteros S. . (2020). Metatranscriptomic analysis to define the Secrebiome, and 16S rRNA profiling of the gut microbiome in obesity and metabolic syndrome of Mexican children. Microb. Cell Factories 19:61. doi: 10.1186/s12934-020-01319-y, 32143621 PMC7060530

[ref20] Gao Y. Zhang G. Jiang S. Liu Y. X. (2024). Wekemo Bioincloud: a user-friendly platform for meta-omics data analyses. iMeta 3:e175. doi: 10.1002/imt2.175, 38868508 PMC10989175

[ref21] Global Cardiovascular Risk Consortium Magnussen C. Alegre-Diaz J. Al-Nasser L. A. Amouyel P. Aviles-Santa L. . (2025). Global effect of cardiovascular risk factors on lifetime estimates. N. Engl. J. Med. 393, 125–138. doi: 10.1056/NEJMoa2415879 40162648 PMC13498409

[ref22] Global Cardiovascular Risk Consortium Magnussen C. Ojeda F. M. Leong D. P. Alegre-Diaz J. Amouyel P. . (2023). Global effect of modifiable risk factors on cardiovascular disease and mortality. N. Engl. J. Med. 389, 1273–1285. doi: 10.1056/NEJMoa2206916 37632466 PMC10589462

[ref23] Guevara-Cruz M. Flores-Lopez A. G. Aguilar‐López M. Sánchez‐Tapia M. Medina‐Vera I. Díaz D. . (2019). Improvement of lipoprotein profile and metabolic endotoxemia by a lifestyle intervention that modifies the gut microbiota in subjects with metabolic syndrome. J. Am. Heart Assoc. 8:e012401. doi: 10.1161/JAHA.119.012401, 31451009 PMC6755842

[ref24] Han Y. Chang H. Huang J. Pan M. Gong J. Huang B. . (2025). Association of 24 conventional and unconventional anthropometric indicators with pre-hypertension or hypertension: a population-based cross-sectional study in Chinese population. Vasc. Health Risk Manag. 21, 859–877. doi: 10.2147/VHRM.S545737, 41140518 PMC12553389

[ref25] Hasan S. Ghani N. Zhao X. Good J. Huang A. Wrona H. L. . (2024). Dietary pyruvate targets cytosolic phospholipase A2 to mitigate inflammation and obesity in mice. Protein Cell 15, 661–685. doi: 10.1093/procel/pwae014, 38512816 PMC11365557

[ref26] Heuser S. K. Li J. Pudewell S. LoBue A. Li Z. Cortese-Krott M. M. (2025). Biochemistry, pharmacology, and *in vivo* function of arginases. Pharmacol. Rev. 77:100015. doi: 10.1124/pharmrev.124.001271, 39952693 PMC12105760

[ref27] Hosomi K. Saito M. Park J. Murakami H. Shibata N. Ando M. . (2022). Oral administration of *Blautia wexlerae* ameliorates obesity and type 2 diabetes via metabolic remodeling of the gut microbiota. Nat. Commun. 13:4477. doi: 10.1038/s41467-022-32015-7, 35982037 PMC9388534

[ref28] Jama H. A. Rhys-Jones D. Nakai M. Yao C. K. Climie R. E. Sata Y. . (2023). Prebiotic intervention with HAMSAB in untreated essential hypertensive patients assessed in a phase II randomized trial. Nat. Cardiovasc. Res. 2, 35–43. doi: 10.1038/s44161-022-00197-4, 39196205

[ref29] Khleborodova A. Gamboa-Tuz S. D. Ramos M. Segata N. Waldron L. Oh S. (2024). Lefser: implementation of metagenomic biomarker discovery tool, LEfSe, in R. Bioinformatics 40:btae707. doi: 10.1093/bioinformatics/btae707, 39585730 PMC11665633

[ref30] Langmead B. Salzberg S. L. (2012). Fast gapped-read alignment with bowtie 2. Nat. Methods 9, 357–359. doi: 10.1038/nmeth.1923, 22388286 PMC3322381

[ref31] Law R. C. Nurwono G. Park J. O. (2024). A parallel glycolysis provides a selective advantage through rapid growth acceleration. Nat. Chem. Biol. 20, 314–322. doi: 10.1038/s41589-023-01395-2, 37537378 PMC10987256

[ref32] Lei Y. Tang L. Liu S. Hu S. Wu L. Liu Y. . (2021). Parabacteroides produces acetate to alleviate heparanase-exacerbated acute pancreatitis through reducing neutrophil infiltration. Microbiome 9:115. doi: 10.1186/s40168-021-01065-2, 34016163 PMC8138927

[ref33] Li R. Huang X. Liang X. Su M. Lai K. P. Chen J. (2021). Integrated omics analysis reveals the alteration of gut microbe-metabolites in obese adults. Brief. Bioinform. 22:bbaa165. doi: 10.1093/bib/bbaa165, 32770198

[ref34] Li X. Ouyang W. Jiang Y. Lin Q. Peng X. Hu H. . (2023). Dextran-sulfate-sodium-induced colitis-ameliorating effect of aqueous *Phyllanthus emblica* L. extract through regulating colonic cell gene expression and gut microbiomes. J. Agric. Food Chem. 71, 6999–7008. doi: 10.1021/acs.jafc.3c00308, 37102314

[ref35] Mancia G. Kreutz R. Brunström M. Burnier M. Grassi G. Januszewicz A. . (2023). 2023 ESH guidelines for the management of arterial hypertension the task force for the management of arterial hypertension of the European Society of Hypertension: endorsed by the International Society of Hypertension (ISH) and the European renal association (ERA). J. Hypertens. 41, 1874–2071. doi: 10.1097/HJH.0000000000003480, 37345492

[ref36] Mizoguchi R. Karashima S. Miyajima Y. Ogura K. Kometani M. Aono D. . (2023). Impact of gut microbiome on the renin-aldosterone system: Shika-machi super preventive health examination results. Hypertens. Res. 46, 2280–2292. doi: 10.1038/s41440-023-01334-7, 37280260

[ref37] Nahm F. S. (2022). Receiver operating characteristic curve: overview and practical use for clinicians. Korean J. Anesthesiol. 75, 25–36. doi: 10.4097/kja.21209, 35124947 PMC8831439

[ref38] Oksanen J. Blanchet F. G. Friendly M. Kindt R. Legendre P. McGlinn D. (2024) Ordination methods, diversity analysis and other functions for community and vegetation ecologists. Available online at: https://github.com/vegandevs/vegan

[ref39] Parizadeh M. Arrieta M. C. (2023). The global human gut microbiome: genes, lifestyles, and diet. Trends Mol. Med. 29, 789–801. doi: 10.1016/j.molmed.2023.07.002, 37516570

[ref40] Prentice R. L. Zhao S. (2021). Regression models and multivariate life tables. J. Am. Stat. Assoc. 116, 1330–1345. doi: 10.1080/01621459.2020.1713792, 34629570 PMC8494047

[ref41] R Development Core Team. (2009). A Language and Environment for Statistical Computing R. R Foundation for Statistical Computing, Vienna, Austria. Available online at: https://www.R-project.org/.

[ref42] Rui W. Li X. Wang L. Tang X. Yang J. (2024). Potential applications of *Blautia wexlerae* in the regulation of host metabolism. Probiotics Antimicrob Proteins. 16, 1866–1874. doi: 10.1007/s12602-024-10274-8, 38703323

[ref43] Sun J. Yang L. Ma C. Yang L. Zhao M. Magnussen C. G. . (2025). Alteration of gut microbiota associated with hypertension in children. BMC Microbiol. 25:282. doi: 10.1186/s12866-025-03999-1, 40340772 PMC12060425

[ref44] Suzuki T. Ogizawa A. Ishiguro K. Nagao A. (2025). Biogenesis and roles of tRNA queuosine modification and its glycosylated derivatives in human health and diseases. Cell Chem Biol. 32, 227–238. doi: 10.1016/j.chembiol.2024.11.004, 39657672

[ref45] Tiwari A. Ika Krisnawati D. Susilowati E. Mutalik C. Kuo T. R. (2024). Next-generation probiotics and chronic diseases: a review of current research and future directions. J. Agric. Food Chem. 72, 27679–27700. doi: 10.1021/acs.jafc.4c08702, 39588716 PMC11660543

[ref46] Tu J. Chen H. Zeng Q. Chen L. Guo Y. Chen K. (2025). Trends in obesity prevalence among adults with hypertension in the United States, 2001 to 2023. Hypertension 82, 498–508. doi: 10.1161/HYPERTENSIONAHA.124.24123, 39758027

[ref47] Vallianou N. G. Kounatidis D. Tsilingiris D. Panagopoulos F. Christodoulatos G. S. Evangelopoulos A. . (2023). The role of next-generation probiotics in obesity and obesity-associated disorders: current knowledge and future perspectives. Int. J. Mol. Sci. 24:6755. doi: 10.3390/ijms24076755, 37047729 PMC10095285

[ref48] Verhaar B. J. H. Collard D. Prodan A. Levels J. H. M. Zwinderman A. H. Bäckhed F. . (2020). Associations between gut microbiota, faecal short-chain fatty acids, and blood pressure across ethnic groups: the HELIUS study. Eur. Heart J. 41, 4259–4267. doi: 10.1093/eurheartj/ehaa704, 32869053 PMC7724641

[ref49] Wang K. Liao M. Zhou N. Bao L. Ma K. Zheng Z. . (2019). *Parabacteroides distasonis* alleviates obesity and metabolic dysfunctions via production of succinate and secondary bile acids. Cell Rep. 26, 222–235.e5. doi: 10.1016/j.celrep.2018.12.028, 30605678

[ref50] Wu F. Guo Y. Wang Y. Sui X. Wang H. Zhang H. . (2024). Effects of long-term fasting on gut microbiota, serum metabolome, and their association in male adults. Nutrients 17:35. doi: 10.3390/nu17010035, 39796469 PMC11722564

[ref51] Wu X. Y. Guo X. Y. Zhang B. Jiang Y. Ye B.-C. (2019). Recent advances of L-ornithine biosynthesis in metabolically engineered *Corynebacterium glutamicum*. Front. Bioeng. Biotechnol. 7:440. doi: 10.3389/fbioe.2019.00440, 31998705 PMC6962107

[ref52] Yu N. Yang Y. Wang G. Wang Y. Feng M. Yang P. . (2025). Investigating the gut microbiota profile in prehypertensive individuals exhibiting phlegm-dampness constitution. Front. Cell. Infect. Microbiol. 15:1507076. doi: 10.3389/fcimb.2025.1507076, 40104285 PMC11913815

[ref53] Yuan Y. Sun W. Kong X. (2022). Relationship between metabolically healthy obesity and the development of hypertension: a nationwide population-based study. Diabetol. Metab. Syndr. 14:150. doi: 10.1186/s13098-022-00917-7, 36229850 PMC9559015

[ref54] Zecheng L. Donghai L. Runchuan G. Yuan Q. Qi J. Yijia Z. . (2023). Fecal microbiota transplantation in obesity metabolism: a meta analysis and systematic review. Diabetes Res. Clin. Pract. 202:110803. doi: 10.1016/j.diabres.2023.110803, 37356723

[ref55] Zhang W. Han N. Zhang T. Qiang Y. Peng X. Li X. . (2022). The spatial features and temporal changes in the gut microbiota of a healthy Chinese population. Microbiol. Spectrum 10:e0131022. doi: 10.1128/spectrum.01310-22, 36453887 PMC9769860

[ref56] Zhang F. Zuo T. Wan Y. Xu Z. Cheung C. Li A. Y. . (2022). Multi-omic analyses identify mucosa bacteria and fecal metabolites associated with weight loss after fecal microbiota transplantation. Innovation 3:100304. doi: 10.1016/j.xinn.2022.100304, 36091491 PMC9460156

