# Supplementary material for: Depletion of Blautia wexlerae and Parabacteroides distasonis in adiposity-related prehypertension
Source: Front Microbiol. 2026 Jun 29;17:1873803. doi: 10.3389/fmicb.2026.1873803 (PMC13357906; doi:10.3389/fmicb.2026.1873803)
Supplement: Supplementary file 1 [file Supplementary_file_1.DOCX]

**Supplementary Table 1 The baseline characteristics of the participants.**

| **Characteristics** | **Total**  (n=1772) | **Overall**  （n=649） | **Normal weight**  (n=383) | **Adiposity**  (n=266) | **FDR** |
| --- | --- | --- | --- | --- | --- |
| Sex, Male, n (%) | 1455(82.1) | 471 (72.6) | 231 (60.3) | 240 (90.2) | <0.001 |
| Age (years) | 55.21 ± 13.72 | 48.37 ± 12.91 | 46.54 ± 12.91 | 51.02 ± 12.45 | <0.001 |
| SBP (mmHg) | 130.54 ± 18.83 | 113.13 ± 9.22 | 111.79 ± 9.51 | 115.06 ± 8.44 | <0.001 |
| DBP (mmHg) | 80.06 ± 10.34 | 70.97 ± 5.73 | 70.25 ± 6.07 | 72.01 ± 5.02 | <0.001 |
| TC (mmol/L) | 4.43 ± 0.88 | 4.45 ± 0.84 | 4.45 ± 0.87 | 4.44 ± 0.79 | 0.789 |
| HDL-C (mmol/L) | 1.22 ± 0.28 | 1.26 ± 0.29 | 1.35 ± 0.30 | 1.13 ± 0.23 | <0.001 |
| LDL-C (mmol/L) | 2.88 ± 0.81 | 2.89 ± 0.77 | 2.85 ± 0.79 | 2.95 ± 0.75 | 0.120 |
| TG (mmol/L) | 1.58 ± 1.02 | 1.45 ± 0.94 | 1.27 ± 0.74 | 1.71 ± 1.11 | <0.001 |
| Diabetes, n (%) | 174(9.8) | 37(5.7) | 13 (3.4) | 24 (9.0) | 0.004 |
| Hyperlipidemia, n (%) | 214(12.1) | 75 (11.6) | 35 (9.1) | 40 (15.0) | 0.029 |
| Prehypertension, n (%) | 776(43.8) | 153 (23.6) | 67 (17.5) | 86 (32.3) | <0.001 |

Continuous variables are presented as mean ± standard deviation (SD), and categorical variables are presented as n (percentage). SBP, systolic blood pressure; DBP, diastolic Blood Pressure; TC, total cholesterol; HDL-C, high-density lipoprotein cholesterol; LDL-C, low-density lipoprotein cholesterol; TG, triglyceride; The Adiposity group refers to individuals with BMI ≥ 24, while the Normal weight group refers to those with BMI < 24. The FDR value for group differences was calculated using the Wilcoxon rank sum test with FDR adjusted for continuous variables, and the Chi-square test (or Fisher's exact test when appropriate) for categorical variables.


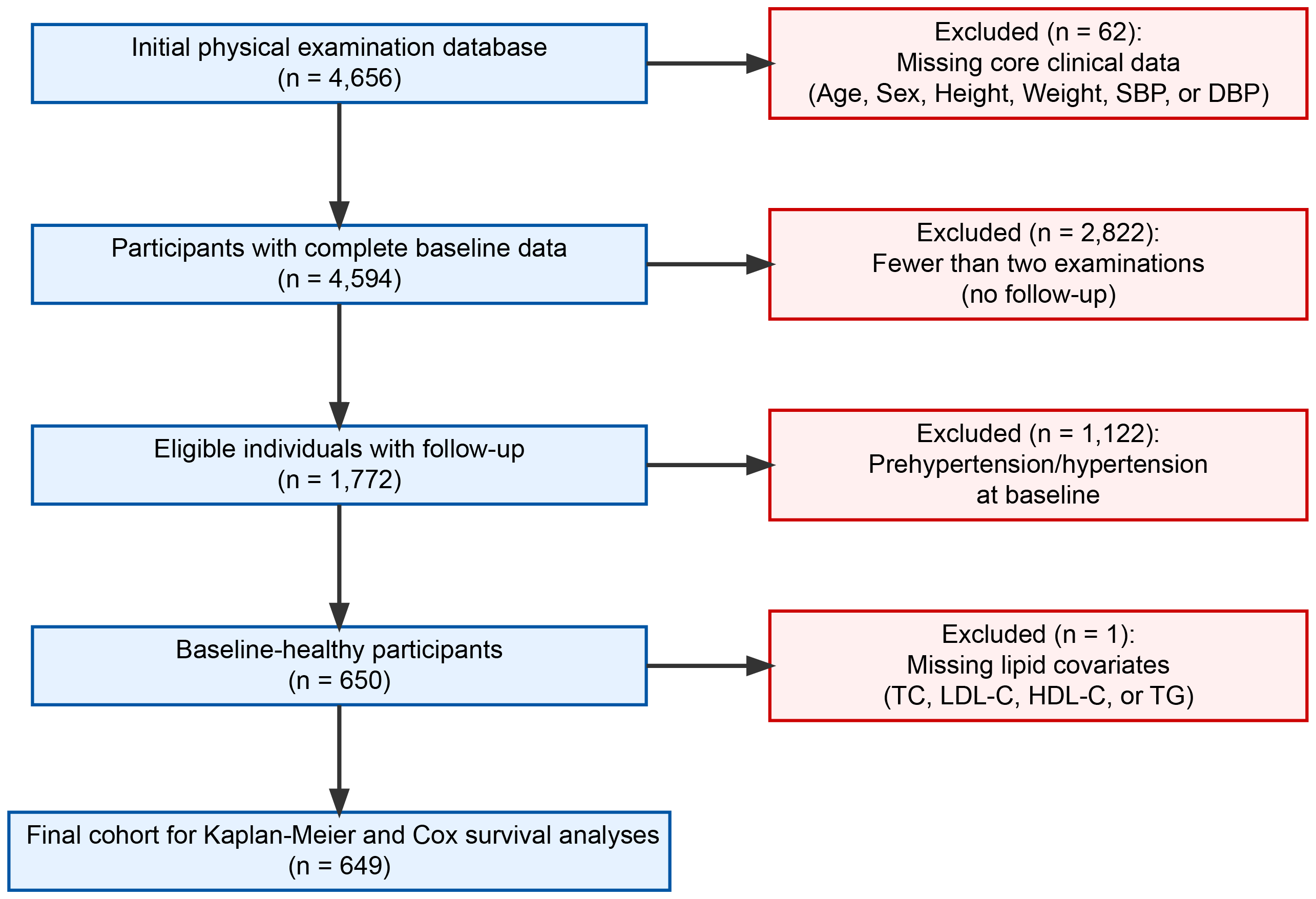


**Supplementary Figure 1 A detailed screening flowchart for retrospective cohort populations.**


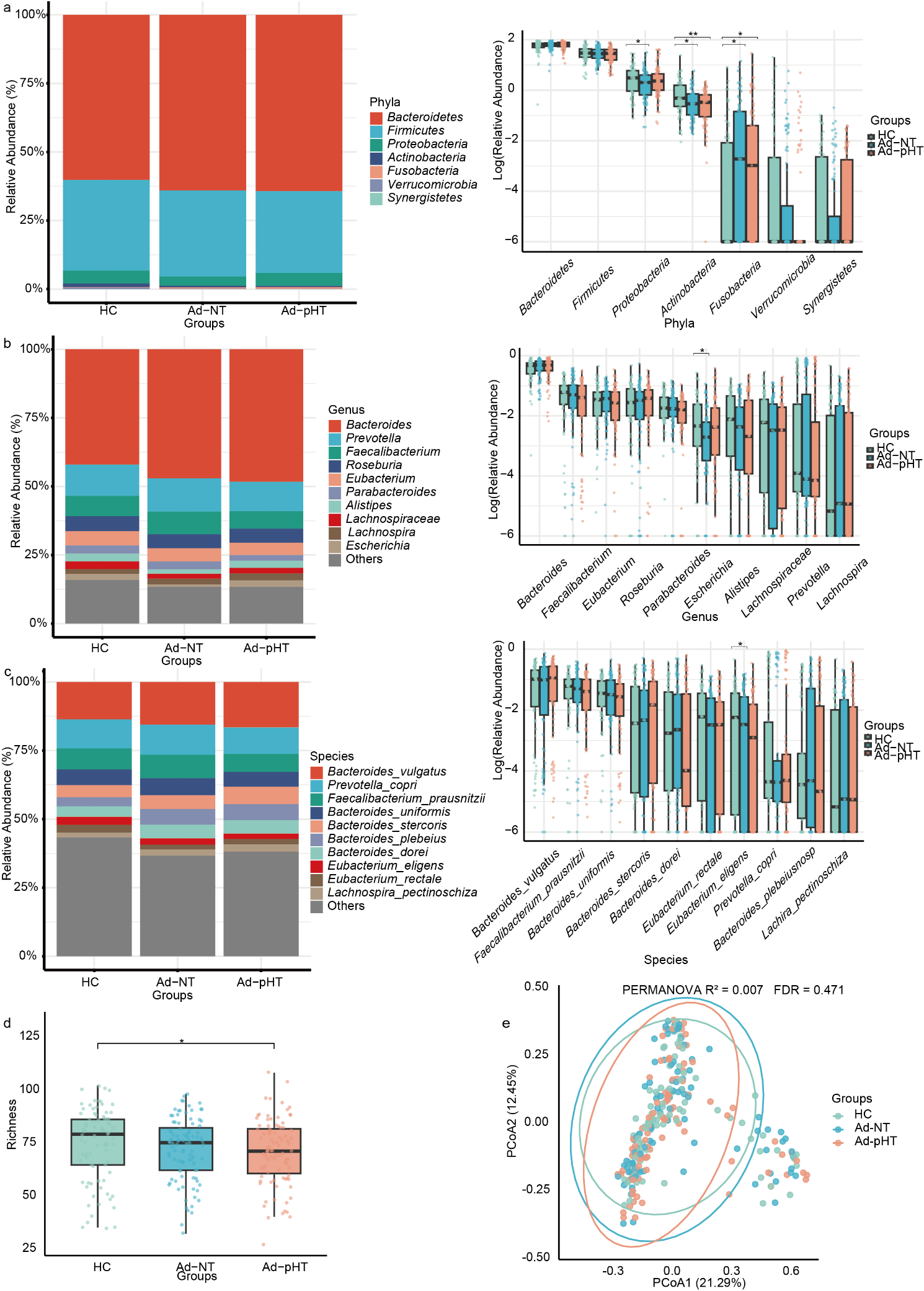


**Supplementary Figure 2 The relative abundance and diversity of gut microbiota.** (a–c) The relative abundance of phyla, genus, and species were compared across three groups using the Wilcoxon rank-sum test with FDR adjusted (*), FDR < 0.05; (**), FDR < 0.01. (d) Richness in HC, Ad-HT, and Ad-pHT was compared using the Wilcoxon rank-sum test with FDR adjusted (*), FDR < 0.05. (e) Beta-diversity. Principal coordinates analysis (PCoA) of gut microbiota revealed differences among three groups, assessed by PERMANOVA (FDR = 0.471).


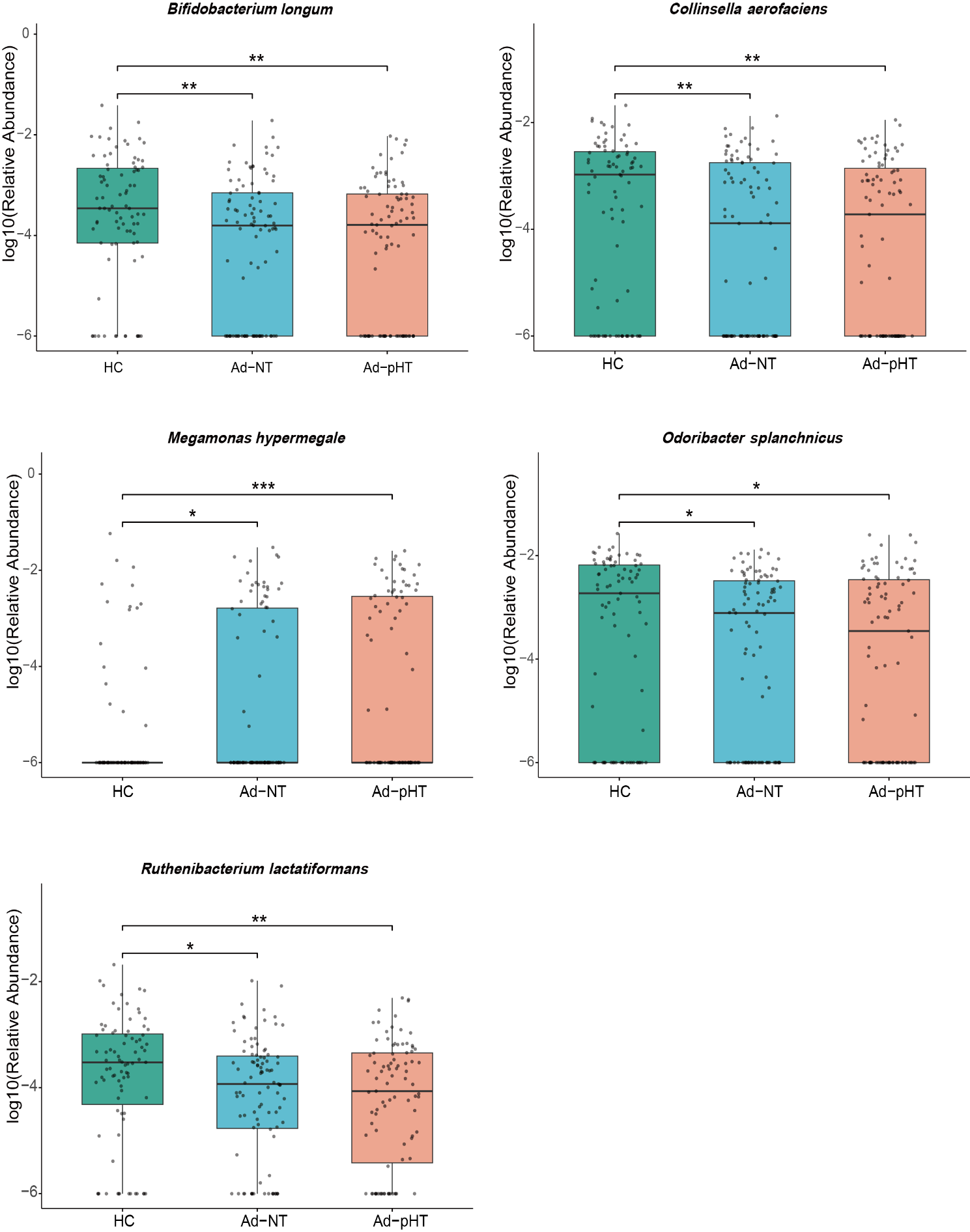


**Supplementary Figure 3 Gut microbiota altered in adiposity.** Relative abundances of species were compared across groups using the Wilcoxon rank-sum test with FDR adjusted (*), FDR < 0.05; (**), FDR < 0.01; (***), FDR < 0.001.

**
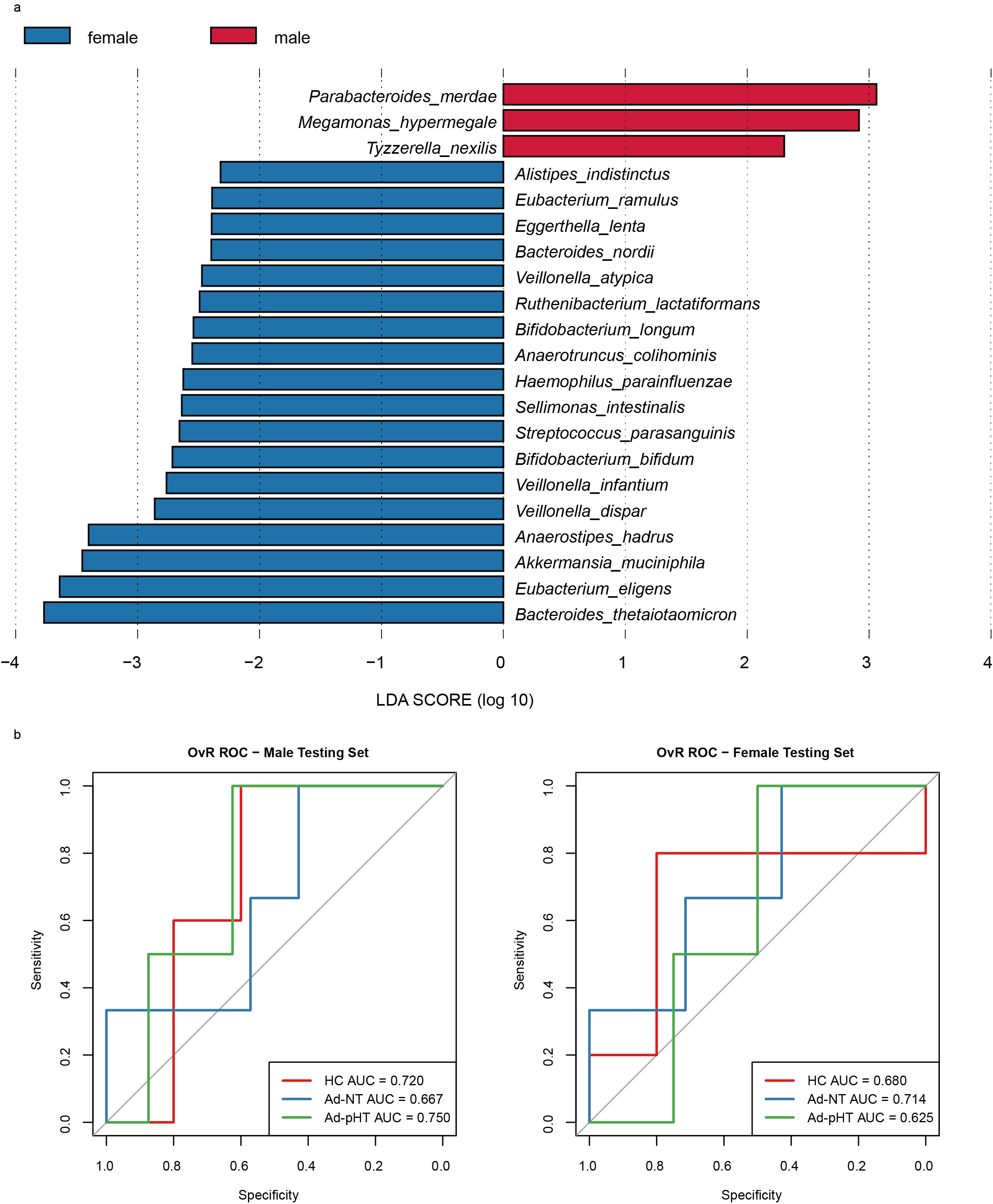
**

**Supplementary Figure 4 Sex-specific differences in gut microbiota. (a)** Different gut microbiota between females and males are determined by LEfSe (LDA > 2, P < 0.05). (b) AUC of the multiclass model with 25 species for males and females.

**
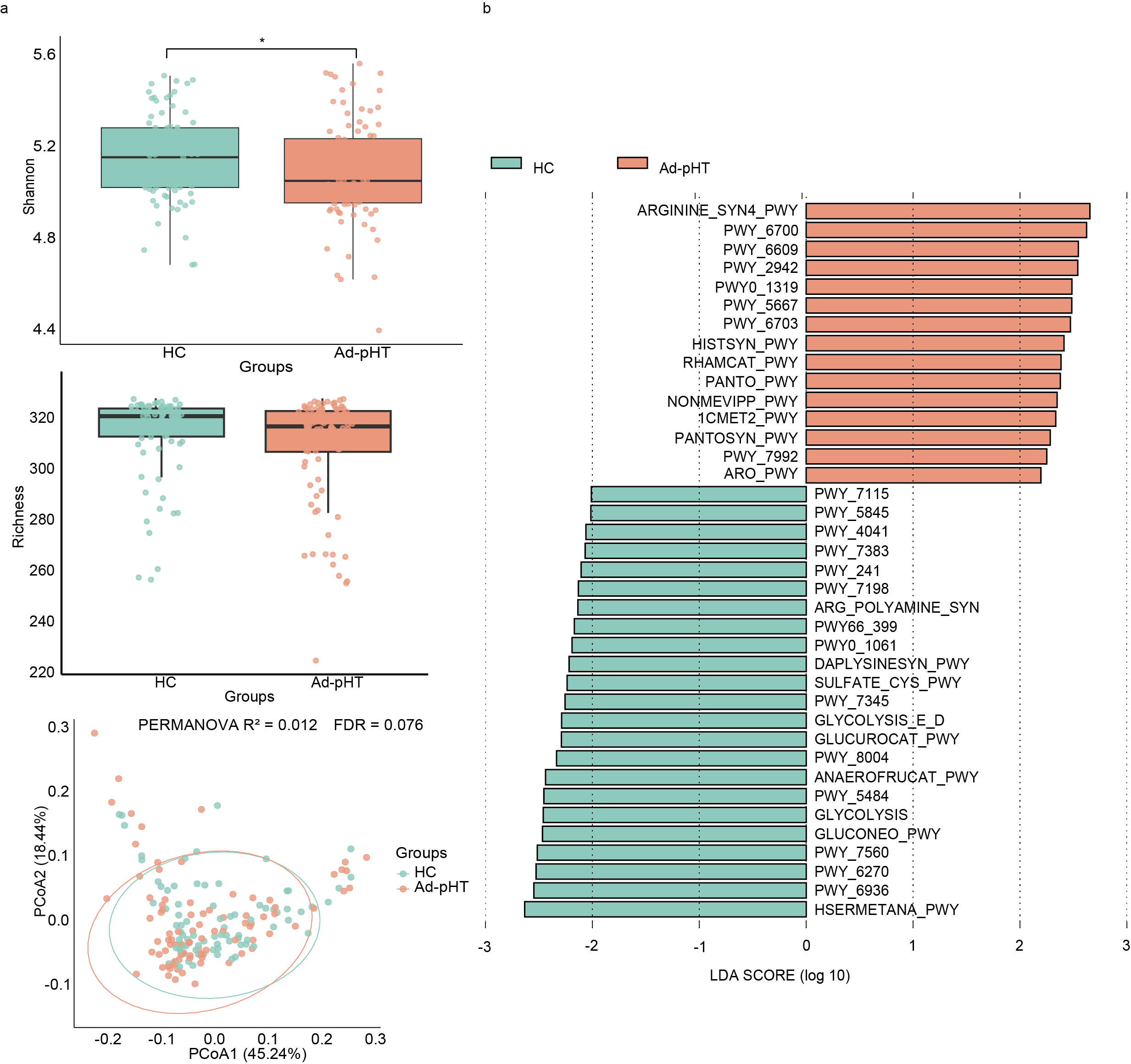
**

**Supplementary Figure 5 Microbial functional alterations in prehypertension with adiposity.** (a) Alpha and beta diversities of the pathway. Shannon diversity and richness were compared using the Wilcoxon rank-sum test with FDR adjusted (*), FDR < 0.05. Principal coordinates analysis (PCoA) of pathways revealed differences between HC and Ad-pHT groups, assessed by PERMANOVA (FDR = 0.076). (b) Different pathways between HC and Ad-pHT are determined by LEfSe (LDA > 2, P < 0.05).


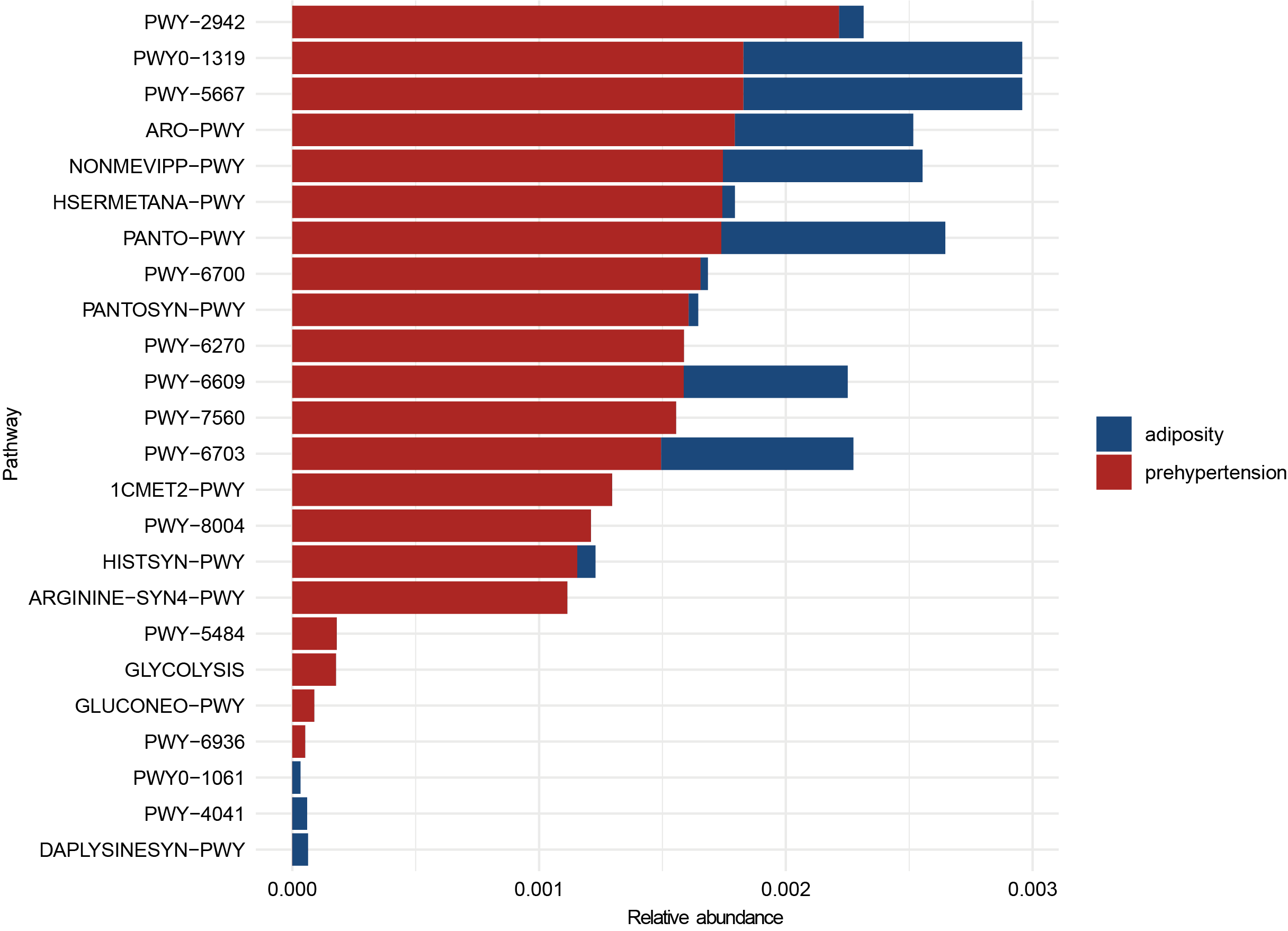


**Supplementary Figure 6 Enrichment analysis of species altered in adiposity and prehypertension in the differential pathway.**


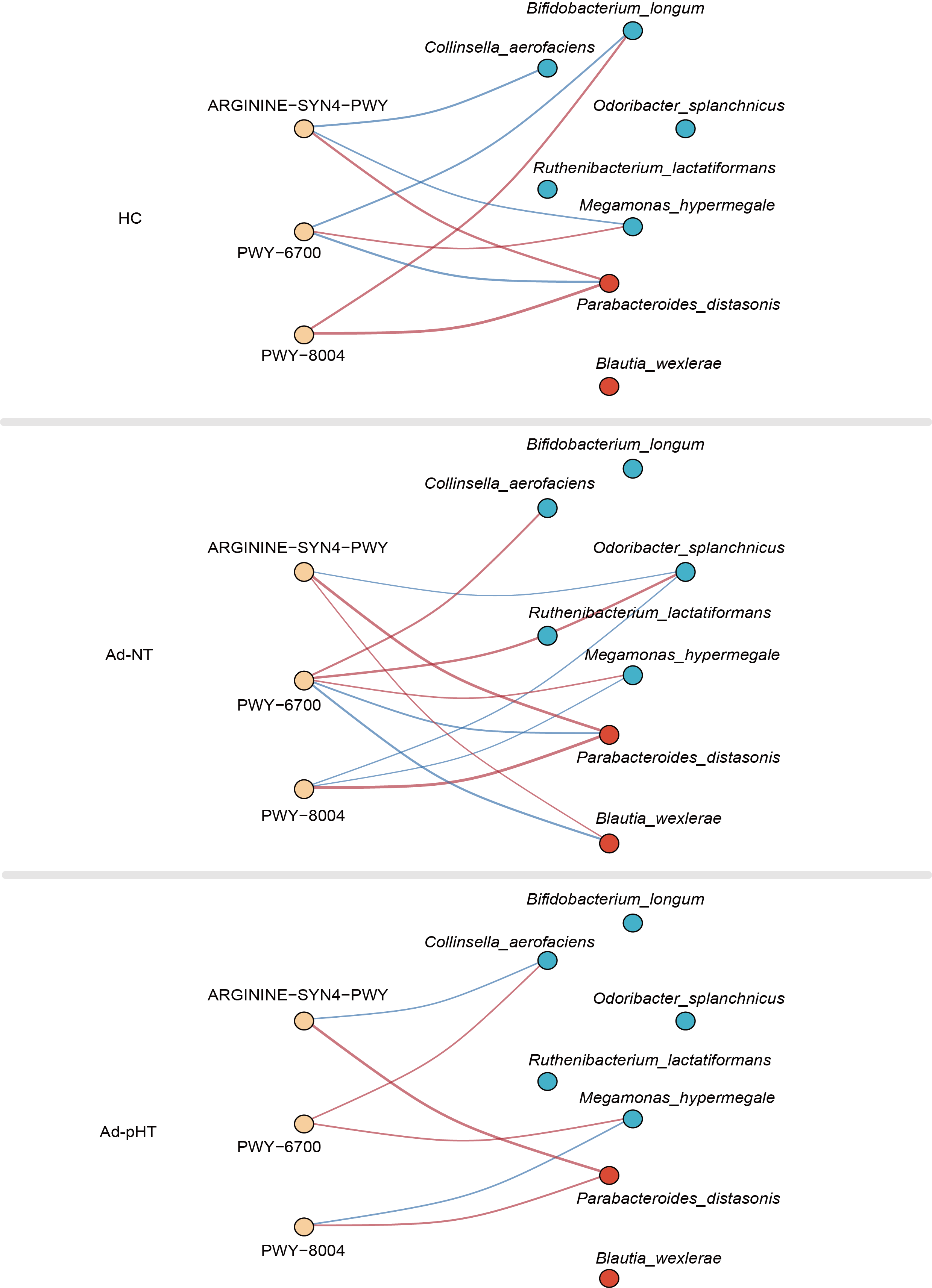


**Supplementary Figure 7 Co-occurrence interactions between three common pathways ( dominated by species specifically perturbed inprehypertension) and seven species ( altered in adiposity and prehypertension).** Partial correlation coefficients and corresponding significance were calculated using Partial correlation analysis, strictly controlling for sex, age, BMI, SBP, DBP, TG, HDL-C, and FBG. P-values were adjusted across the matrix using the False Discovery Rate (FDR) method to control for multiple comparisons (FDR < 0.1). Edge colors indicated correlation direction: red for positive and blue for negative Spearman correlations. The red nodes were species negatively associated with prehypertension, the blue nodes were species altered in adiposity, and the yellow nodes were pathways dominated by species specifically perturbed inprehypertension.
